# Social conflict imagery induces stress, social and physical pain: sex differences in autonomic and affective responses and pathways to resilience and wellbeing

**DOI:** 10.3389/fpsyg.2026.1738699

**Published:** 2026-08-05

**Authors:** Jae Chan Choi, In-Ho Choi, KeeSam Jeong, Jee Yeon Kim, Jun-Ho Choi

**Affiliations:** 1 Department of Counseling Psychology, Graduate School of Seoul Cyber University, Seoul, Republic of Korea; 2 Department of Anesthesiology and Pain Medicine, Yonsei University Wonju College of Medicine, Wonju, Republic of Korea; 3 Cham Psychology Brain Health Institute, Seoul, Republic of Korea; 4 Department of Cartoon and Animation, Cheongju University, Cheongju-si, Republic of Korea; 5 Medicore Ltd., Seongnam, Republic of Korea; 6 Department of Counseling Psychology, Seoul Cyber University, Seoul, Republic of Korea; 7 Jun Neuro-Psychology & Counseling Institute, Seoul, Republic of Korea

**Keywords:** autonomic nervous system, loneliness (social pain), physical pain, sex differences, social conflict, stress

## Abstract

**Introduction:**

This study investigated how viewing images depicting male-female conflict influences emotional distress, autonomic regulation, and pain.

**Methods:**

Sixty-five adults completed pre-experiment questionnaires and were exposed to a plus sign (baseline condition) and conflict images (conflict condition). Emotional responses and heart rate variability (HRV) were assessed during the experiment, and pain was evaluated following each condition. HRV analyses included absolute and normalized spectral measures. High-frequency HRV (HF-HRV) primarily reflected parasympathetic activity, whereas low-frequency (LF)-related indices reflected mixed and relative autonomic influences rather than pure sympathetic activity.

**Results:**

Exposure to conflict images produced psychological distress and physiological dysregulation, characterized by increased stress, anxiety, fear, state loneliness, negative societal perceptions, and pain, accompanied by reduced happiness, empathy, and positive societal perceptions. Conflict exposure significantly reduced absolute parasympathetic activity, as indicated by lower HF and lnHF, whereas absolute LF power remained unchanged. Thus, the principal autonomic response to conflict reflected selective parasympathetic withdrawal rather than increased absolute LF activity. Age-adjusted analyses revealed sex-specific patterns of autonomic and emotional responses. Women exhibited higher normalized HF-HRV (HF norm), happiness, empathy, and positive societal perceptions, together with lower negative societal perceptions. In contrast, men exhibited higher normalized LF-HRV (LF norm) and LF/HF ratios, reflecting greater relative LF predominance and differences in autonomic quality rather than increased sympathetic activation. Significant sex × age interactions were observed for stress, happiness, and empathy, indicating divergent age-related trajectories between men and women, with women showing declines in these variables with increasing age. Regression analyses further showed that conflict-related stress was positively associated with trait loneliness, depression, anxiety, and LF-related indices, whereas higher root mean square of successive differences (RMSSD), reflecting better parasympathetic regulation, was associated with lower stress. Post-conflict pain was positively associated with female sex, stress, and LF-related indices and negatively associated with emotional resources.

**Discussion:**

Social conflict imagery elicited selective parasympathetic withdrawal, autonomic spectral redistribution, psychological distress, and increased pain, particularly among psychologically vulnerable individuals. Conversely, adaptive autonomic regulation, emotional resources, and social support promoted resilience and enhanced wellbeing. These findings highlight psychophysiological and psychosocial pathways through which vulnerability and resilience shape stress, wellbeing, and pain-related outcomes under social conflict. The ecological validity of these findings warrants further investigation.

## 1 Introduction

Interpersonal conflict is a pervasive and often unavoidable aspect of human relationships. Conflict between men and women is particularly salient because it frequently evokes intense emotions and influences psychological wellbeing, relationship functioning, and resilience (Fincham and Beach, 1999; Gottman and Levenson, 1992). Recent studies further indicate that the manner in which individuals perceive and regulate conflict within close relationships is closely associated with overall wellbeing and adaptive functioning (Gallo et al., 2026; Zhang and Chen, 2025a). Importantly, interpersonal conflict is not restricted to romantic relationships but occurs across diverse social contexts and represents a fundamental component of human interaction (Moseti et al., 2025; Welch et al., 2022). Because social conflict is both common and emotionally meaningful, understanding its psychological and physiological consequences is essential for elucidating mechanisms underlying stress and wellbeing.

Notably, social conflict does not need to be directly experienced to produce emotional consequences. Visual representations of emotionally salient social events can evoke robust affective responses in observers (Lang et al., 1997; Saarimaki, 2021; Theodosiou et al., 2026). Neuroimaging studies have shown that observing emotionally meaningful situations recruits neural systems involved in affective processing and social cognition that substantially overlap with those engaged during direct experiences (Jackson et al., 2005; Lamm et al., 2011; Singer et al., 2004; Zhang and Chen, 2025b). Likewise, observational exposure to relational discord can activate stress-response systems resembling those elicited during firsthand interpersonal conflict (Gallistl et al., 2025; Sbarra et al., 2021). Consequently, images depicting male–female conflict provide an ecologically valid approach for experimentally inducing social stress under controlled laboratory conditions. Exposure to interpersonal conflict is consistently associated with heightened psychological distress, including stress, anxiety, fear, loneliness, and negative social cognitions (Bierstetel and Slatcher, 2020; Dovala et al., 2018; Merrill and Afifi, 2017; Yueyan et al., 2025). In addition, relational stress has been associated with diminished empathy and less positive perceptions of others and society (Moreno et al., 2023). Collectively, these findings suggest that social conflict is not merely a transient emotional event but a potent social stressor capable of disrupting emotional and interpersonal functioning.

The emotional consequences of social conflict may also extend to experiences of pain. Contemporary models conceptualize pain as a multidimensional phenomenon encompassing sensory, emotional, cognitive, and social components (Williams and Craig, 2016). Pain perception is strongly influenced by contextual and psychological factors, including emotional state, attention, and expectations (Bushnell et al., 2013). Negative emotions generally amplify pain experiences, whereas positive emotional states attenuate pain perception and facilitate endogenous pain inhibition (Lang-Illievich et al., 2024; Villemure and Bushnell, 2009). Beyond emotional states, social experiences themselves are important determinants of pain. Individuals experiencing loneliness are substantially more likely to report physical pain, health problems, and psychological distress (Macchia and Fett, 2025). Moreover, social pain and physical pain share partially overlapping neurobiological mechanisms (Sturgeon and Zautra, 2016). Neuroimaging studies have demonstrated common involvement of the anterior cingulate cortex and insula, regions critically implicated in both emotional processing and pain perception (Yu et al., 2025). Loneliness, anxiety, and depression have also been identified as factors that exacerbate both acute and chronic pain (Blyth et al., 2007; Linton and Shaw, 2011). Together, these findings suggest that social conflict may simultaneously influence emotional distress, social pain, and physical pain through interconnected affective and neurobiological pathways.

Because responses to social conflict involve both subjective experiences and physiological regulation, objective assessment of autonomic nervous system activity is essential. Heart rate variability (HRV) is a well-established marker of autonomic function and stress regulation (Immanuel et al., 2023; Kim et al., 2018). Stress and threat can alter autonomic regulation, often involving reductions in parasympathetic activity and changes in the relative distribution of autonomic resources. High-frequency HRV (HF-HRV) and the root mean square of successive R-R interval differences (RMSSD) primarily reflect parasympathetic activity and are commonly associated with emotional regulation and adaptive stress responses (Appelhans and Luecken, 2006; Shaffer and Ginsberg, 2017). Higher parasympathetic activity has also been linked to better sleep quality, lower fatigue, and reduced daily stress, supporting its role as an indicator of autonomic recovery and wellbeing (Hannon et al., 2025). Although low-frequency HRV (LF-HRV), normalized HRV measures, and the LF/HF ratio have traditionally been interpreted as markers of sympathetic activity and sympathovagal balance, contemporary evidence indicates that these indices reflect complex interactions between sympathetic and parasympathetic influences and should therefore be interpreted cautiously (Berntson et al., 2008; Quigley et al., 2024). Importantly, normalized spectral measures are mathematically dependent on proportional allocation and cannot determine whether individual spectral components have increased or decreased simultaneously. Accordingly, the present study distinguished between autonomic quantity, indexed by absolute spectral power (e.g., HF and LF power), and autonomic quality, indexed by relative spectral composition (e.g., HF norm, LF norm, and LF/HF ratio). Examining both absolute and relative HRV measures may therefore provide a more comprehensive understanding of psychophysiological responses to socially induced stress and pain.

Importantly, substantial sex differences exist in emotional, autonomic, and pain responses. Women generally exhibit greater pain sensitivity and report pain that is more frequent, severe, and widespread than men (Bartley and Fillingim, 2013; Failla et al., 2024; Fillingim et al., 2009; Smith et al., 2020). Sex differences in pain are evident not only in physiological sensitivity but also in the emotional distress accompanying painful experiences, with women generally reporting greater pain-related emotional distress than men (Puto et al., 2024). Conversely, women generally exhibit greater parasympathetic activity, as indicated by higher HF-HRV, than men (Koenig and Thayer, 2016). Studies of psychological stress indicate sex-specific patterns of autonomic regulation. Stress-induced HF power significantly decreases in men but increases in women (Adjei et al., 2018). Furthermore, higher HF-HRV is more strongly associated with perceived social support under stress in women, suggesting sex differences in parasympathetic regulation under stress (Kvadsheim et al., 2022). These findings suggest that sex may substantially shape how individuals emotionally and physiologically respond to social conflict.

Despite considerable evidence linking interpersonal conflict, emotional distress, pain, and autonomic regulation, relatively few studies have simultaneously examined these domains in response to experimentally induced social conflict. In particular, the psychophysiological pathways through which viewing male–female conflict images influences emotional distress, social pain, physical pain, and autonomic activity remain poorly understood. Therefore, the present study investigated the effects of viewing male–female conflict images on emotional responses, autonomic nervous system activity indexed by HRV, and physical pain using a within-subject experimental design. We hypothesized that exposure to conflict images would increase stress, state loneliness, anxiety, fear, negative societal thoughts, and pain while reducing emotional resources, empathy, positive societal perceptions, and happiness. We further hypothesized that conflict exposure would be accompanied by altered autonomic regulation, that emotional and physiological responses would differ by sex, and that conflict-related stress, pain, loneliness, and happiness would be associated with autonomic activity and pre-existing psychological vulnerabilities and protective factors. Accordingly, we formulated the following hypotheses regarding the emotional, autonomic, and pain-related consequences of viewing social conflict images:

*H1*: Exposure to social conflict images (conflict condition) is expected to significantly increase participants’ stress, state loneliness, anxiety, fear, negative thoughts about society, and pain scores compared to baseline, while simultaneously decreasing emotional resources (emotional bank account), positive thoughts about society, empathy, and happiness.

*H2*: During social conflict exposure, HRV indices are expected to reflect decreased parasympathetic activity (HF, lnHF, RMSSD) together with changes in LF-related indices, reflecting stress-induced alterations in autonomic regulation rather than direct sympathetic activation.

*H3*: Sex differences are expected to emerge in both emotional and physiological responses to social conflict. Specifically, women are expected to exhibit higher HF-HRV, greater happiness and empathy, more positive societal thoughts, and higher perceived pain than men.

*H4*: Stress scores under social conflict are expected to be associated with lower parasympathetic regulation, reflected by lower RMSSD, higher LF-related indices, and pre-existing psychological vulnerabilities, including trait loneliness (UCLA trait loneliness), depression, and Beck anxiety.

*H5*: Post-conflict pain scores are expected to be positively associated with female sex, stress, number of family members, and sympathetic activity (lnLF), and negatively associated with emotional resources (emotional bank account) and negative social thoughts.

*H6*: Loneliness during the conflict condition is expected to reflect both vulnerability and protective processes, being positively associated with trait loneliness and negatively associated with affectionate support and sleep duration.

*H7*: Happiness during the conflict condition is expected to reflect resilience-related processes, being negatively associated with depressive symptoms and positively associated with emotional and social resources.

Collectively, these hypotheses were designed to elucidate the psychophysiological pathways linking social conflict exposure to emotional distress, autonomic regulation, pain perception, and sex-specific responses, all of which contribute to individual differences in vulnerability and resilience.

## 2 Materials and methods

### 2.1 Participants

The experiment was conducted in accordance with the Declaration of Helsinki and approved by the Medical Ethics Committee of Yonsei University, Wonju College of Medicine (CR124036). Informed consent was obtained from all participants. Sixty-five heterosexual volunteers from South Korea (30 men and 35 women; mean age = 48.92 years, SD = 11.69) were recruited and compensated for their participation. All participants provided written informed consent, acknowledging that (1) the methods and procedures were clearly explained, and (2) they could withdraw from the experiment at any time. Participants were instructed to avoid alcohol for 24 h and caffeine for 2 h prior to the study. Based on a significance level of 0.05, a power of 0.8, and assuming a small effect size (Cohen’s *d* = 0.31) for the increase in pain due to the conflict (stress) condition, the required sample size was calculated to be 65 (64.33).

### 2.2 Psychometric measures

Participants completed the following questionnaires on the day before the study: the UCLA Loneliness Scale Version 3, Beck Anxiety Inventory (BAI), and Patient Health Questionnaire-9 (PHQ-9). To assess trait and state loneliness (Wiseman, 1997) prior to the experiment, we employed the Korean-translated version (Jin and Hwang, 2019) of the UCLA Loneliness Scale Version 3 (Russell, 1996), which evaluates subjective feelings of loneliness and social isolation and has been extensively validated since its development (Russell, 1996) and continues to demonstrate strong psychometric properties in recent cross-cultural studies (Zeas-Sigüenza et al., 2022). The scale comprises 20 items rated on a 4-point scale (1 = never, 2 = rarely, 3 = sometimes, 4 = often), with higher scores indicating greater loneliness. In the subsequent sections, the score from the UCLA Loneliness Scale Version 3 will be referred to as the trait loneliness score to distinguish it from the state loneliness score assessed during the experiment (Roddick and Chen, 2021). Anxiety was assessed using the Korean version of the Beck Anxiety Inventory (Sung Pil and Zoung Soul, 1997). The BAI has demonstrated good reliability and validity in Korean populations, with evidence supporting its internal consistency, test–retest reliability, and factorial structure in community-dwelling adult (Lee et al., 2016). The BAI (Beck et al., 1988) is a self-report tool for evaluating anxiety in adults and adolescents, comprising 21 items describing common anxiety symptoms. Participants rate the extent to which each symptom has bothered them over the past week on a scale from 0 (not at all) to 3 (severely), with total scores summed to indicate overall anxiety severity. The BAI has been extensively validated since its development (Beck et al., 1988) and continues to demonstrate strong psychometric properties in recent studies across diverse and high-stress populations, including refugee samples (Mazhak and Sudyn, 2024). In the subsequent sections, the score obtained from the BAI will be referred to as the Beck anxiety score to distinguish it from anxiety assessed during the experiment. Depression was assessed using the Korean version (Park et al., 2010) of the Patient Health Questionnaire-9 (PHQ-9) (Spitzer et al., 1999), a self-report tool designed to screen for depression and assess its severity. The PHQ-9 comprises nine items corresponding to the DSM-IV diagnostic criteria for major depressive disorder. Respondents indicate how often they have experienced each symptom over the past 2 weeks on a 4-point scale: 0 (not at all), 1 (several days), 2 (more than 1 week), and 3 (nearly every day), with total scores ranging from 0 to 27. The PHQ-9 has been extensively validated since its development (Spitzer et al., 1999) and continues to demonstrate strong reliability and validity across diverse populations in recent studies (Fonseca-Pedrero et al., 2023; Kim et al., 2023). In the subsequent sections, the score obtained from the PHQ-9 will be referred to as the PHQ-9 total score to distinguish it from depression assessed during the experiment.

To assess social support prior to the experiment, the Korean version of the Medical Outcomes Study Social Support Survey (MOS-SSS) (Lim et al., 2003), adapted from the original scale by Sherbourne and Stewart (1991), was used. This measure continues to be widely applied in recent research on social support and health outcomes (Jin and Hong, 2023). The MOS-SSS comprises 19 items across four subdomains: emotional/informational support (8 items), tangible support (4 items), affectionate support (3 items), and positive social interaction (4 items), with total scores ranging from 19 to 95; higher scores indicate greater social support. Emotional/informational support assesses the availability of others who provide empathy, encouragement, and advice during difficult times. Tangible support reflects the level of practical or material assistance received from others. Affectionate support measures emotional closeness and affectionate relationships, while positive social interaction refers to engaging in social activities that offer companionship and mutual enjoyment, such as spending time with supportive friends or family.

### 2.3 Experimental procedure

During both the baseline and conflict conditions, participants sat comfortably with their heads supported and viewed a 354.85 × 227.97 mm computer screen positioned approximately 60 cm from their eyes, with the center of the screen located 10–20° below eye level. In the present study, the baseline was defined operationally as a 3-min period during which participants fixated on a centrally presented plus sign (“+”) displayed on the computer screen (Krempel et al., 2025). This definition was adopted to standardize participants’ visual attention and minimize variability in eye position across conditions. The baseline was conducted first to minimize carryover effects (Bahlinger et al., 2022; Cribbet et al., 2020) and to provide an uncontaminated reference for HRV (Laborde et al., 2017), given that baseline vagal tone serves as a critical physiological reference for interpreting subsequent pain-related responses (Gozansky et al., 2026). In the conflict condition, participants subsequently viewed a 3-min series of 60 photographs depicting men and women glaring at each other with grim facial expressions, representing social threat. The phrase “Social Conflict” was displayed continuously at the top of the screen to reinforce the perception of conflict. Images were generated using Midjourney, a generative artificial intelligence program developed by the San Francisco–based independent research lab Midjourney, Inc., which produces images from natural language prompts (Rose, 2022). Recent advances in generative artificial intelligence have demonstrated the capacity to produce high-quality and contextually adaptable outputs across domains (Bubeck et al., 2023). Accordingly, Midjourney was used in the present study to create images that are more culturally appropriate for East Asian participants, rather than relying on commonly used Western-based stimuli. All images were rated by 12 independent raters (who did not participate in the main experiment) using 9-point scales, confirming high conflict intensity (*M* = 7.42, SD = 0.51) and negative emotional valence (*M* = 2.17, SD = 0.72; 1 = very negative).

### 2.4 Pain measurement

Physical pain was measured twice: once after the baseline and once after the conflict condition. Pain was assessed using a 100-g von Frey hair as a mechanical stimulus. Punctate mechanical stimuli were applied by placing a 100-g von Frey hair (North Coast Medical, Morgan Hill, CA, United States) perpendicularly onto the dorsal mid-phalanx of the left middle finger and gradually increasing pressure until the hair bent slightly (Beissner et al., 2010; Rowbotham and Wallace, 2020). Previous research has shown that a 26-g von Frey filament produces a mildly noxious pin-like sensation (Rowbotham and Wallace, 2020). In recent microneurography research (Ng et al., 2024), lower von Frey forces (e.g., ≤ 20 mN) evoke minimal or inconsistent responses in C nociceptors, whereas firing rates increase more reliably at higher forces (≥ 100 mN). In addition, both A and C nociceptors exhibit progressively increased activation from approximately 100 to 3,000 mN, indicating a force-dependent encoding of noxious mechanical stimuli. Based on this evidence, a higher von Frey force (100 g; ≈ 980 mN) was selected to ensure a clearly perceivable nociceptive stimulus while avoiding excessive discomfort, thereby providing a consistent and sufficiently salient mechanical pain stimulus across participants. Based on previous findings showing that pain perception is significantly reduced when inter-stimulus intervals are shorter than 60 s (Schestatsky et al., 2007), an interval of 70 s was used between stimulations. This approach is further supported by recent evidence demonstrating substantial inter-individual variability in pain habituation, with repeated nociceptive stimulation influenced by both peripheral adaptation and endogenous pain modulation processes (De Schoenmacker et al., 2024). Pain intensity was rated on a numerical rating scale (NRS) from 0 to 100, where 0 represents “no pain” and 100 represents “the worst pain imaginable.” Final pain intensity for each condition (baseline and conflict) was calculated as the mean of ratings obtained during three consecutive stimulations.

### 2.5 Numerical rating scale

Scores for state loneliness, anxiety, stress, fear, positive and negative thoughts about society, empathy, happiness, and the emotional bank account were assessed twice—once after the baseline and once after the conflict condition—using a numerical rating scale for each condition. While the concept of an “emotional bank account” traditionally refers to an imaginary account representing the level of trust in a relationship (Tranter et al., 2018), in the present study, it was adapted and operationalized to capture participants’ sense of emotional investment or replenishment across experimental conditions. Specifically, participants’ experiences of feeling emotionally invested or replenished—conceptually analogous to “making a deposit” into their emotional bank account—were quantified using the Emotional Bank Account Score. Scores on the numerical rating scale ranged from 0 (“not at all”) to 100 (“the most”).

### 2.6 HRV measurement

HRV indices were extracted twice: once during a 3-min baseline and once during a 3-min conflict condition. HRV was measured on an empty stomach, with participants in a seated position, for 3 min using an HRV analyzer (SA-3000NEW, Medicore, Seoul, Korea). This device has been used in several peer-reviewed studies investigating autonomic function and HRV in ophthalmologic, metabolic, and psychological contexts (Jung et al., 2021; Lee et al., 2025; Shin et al., 2020).

HRV analysis can be broadly categorized into time- and frequency-domain analyses. Time-domain measures include mean heart rate, the standard deviation of normal-to-normal intervals (SDNN), and the root mean square of successive differences (RMSSD). SDNN is mathematically similar to total spectral power and provides an estimate of overall autonomic activity. RMSSD primarily reflects parasympathetic influences (Kleiger et al., 2005; Laborde et al., 2017; Thayer and Lane, 2000), is considered an index of vagal tone, and is highly correlated with HF-HRV (Kleiger et al., 2005). More broadly, HRV indices such as RMSSD and HF-HRV are widely used as noninvasive markers of cardiac autonomic regulation and overall physiological health, with reduced HRV consistently associated with a range of clinical conditions, including cardiovascular disease, depression, and cancer-related fatigue (Lucius, 2023).

Frequency-domain analysis transforms the R-R interval series into its constituent spectral components using Fourier transformation. Short-term HRV indices include high frequency (HF), low frequency (LF), and very low frequency (VLF). The HF component (0.15–0.40 Hz), influenced by respiratory sinus arrhythmia, primarily reflects parasympathetic (vagal) activity. The LF component (0.04–0.15 Hz), associated with Mayer-wave oscillations in arterial pressure, reflects complex interactions between sympathetic and parasympathetic influences rather than pure sympathetic activity (Berntson et al., 2008; Quigley et al., 2024). Log-transformed LF (lnLF) and HF (lnHF) represent logarithmically transformed absolute spectral power. VLF (0.0033–0.04 Hz) reflects slower regulatory processes involving multiple physiological mechanisms and should not be interpreted as a direct index of sympathetic activity.

To facilitate interpretation, the present study distinguished between autonomic quantity and autonomic quality. Absolute spectral measures (HF, LF, lnHF, and lnLF) were considered indices of autonomic quantity, representing the absolute magnitude of autonomic activity. In contrast, normalized measures (HF norm, LF norm, and the LF/HF ratio) were considered indices of autonomic quality, representing the relative distribution and allocation of autonomic resources within the overall autonomic space. Because normalized spectral measures are mathematically dependent on proportional allocation, they cannot determine whether both spectral components have increased or decreased simultaneously. Accordingly, LF-related and normalized HRV indices were interpreted within the context of absolute spectral power and relative spectral composition rather than as direct measures of sympathetic activation or withdrawal.

HRV signal processing followed the recommendations of the Task Force of the European Society of Cardiology and the North American Society of Pacing and Electrophysiology (“Heart rate variability: standards of measurement, physiological interpretation and clinical use. Task Force of the European Society of Cardiology and the North American Society of Pacing and Electrophysiology,” Circulation, 1996). Recent evidence further emphasizes the importance of accounting for physiological, pathological, lifestyle, and environmental factors that influence HRV, including age, sex, disease status, physical activity, and external stressor (Sammito et al., 2024a). Contemporary guidelines likewise highlight the necessity of standardized measurement procedures, rigorous data quality control, and appropriate contextualization of HRV indices in laboratory and applied settings (Sammito et al., 2024b). Autonomic nervous system responses were evaluated using HRV in both baseline and conflict conditions. To reduce artifacts, ectopic beats unrelated to autonomic activity (e.g., arrhythmias) were removed. Beats with consecutive R-R interval differences exceeding ± 30% were considered outliers and replaced via linear interpolation. Participants with more than two ectopic beats per minute were excluded from the study to ensure accurate HRV analysis.

### 2.7 Statistical analyses

Data from five participants with technical errors during HRV measurement and four participants with more than two ectopic beats per minute were excluded from the HRV analysis but included in the overall analysis of all participants (*N* = 65). Thus, HRV analyses were conducted on data from 56 participants with valid HRV recordings. Statistical analyses were performed using SPSS version 29 (SPSS Inc., Chicago, IL, United States). Paired-sample *t*-tests were conducted to examine differences between the baseline and conflict conditions. To statistically control for age-related confounding, pre-experimental measures were examined using analysis of covariance (ANCOVA), with age included as a covariate (Khasawneh, 2024). Adjusted outcomes are presented as estimated marginal means with standard errors. Furthermore, a multivariate analysis of covariance (MANCOVA) was performed to investigate sex-based differences in emotional, pain-related, and autonomic responses across baseline and conflict conditions, while accounting for age (Lemire et al., 2024). To further interpret significant sex × age interactions, follow-up regression analyses were conducted separately for men and women to examine the associations between age and stress, happiness, and empathy. Multiple linear regression analyses were conducted to identify psychological and physiological predictors of stress, state loneliness, and happiness during the conflict condition, as well as post-conflict pain. In the first model (*n* = 65), the dependent variable was stress during the conflict condition, with depression score and trait loneliness score (assessed using the UCLA Loneliness Scale) entered as independent variables. In the second model (*n* = 56), two physiological indicators of autonomic nervous system activity—RMSSD and LF-HRV, both measured during the conflict condition—were added to the psychological predictors from the first model. In the third model (*n* = 65), post-conflict pain was entered as the dependent variable, and sex (0 = male, 1 = female), number of family members, emotional bank account during the conflict condition, stress during the conflict condition, and negative societal thoughts during the conflict condition were entered as independent variables. In the fourth model (*n* = 56), lnLF during the conflict condition was added to the set of predictors in the third model. In the fifth model (*n* = 65), the dependent variable was state loneliness during the conflict condition, with trait loneliness, affectionate support, and sleep duration entered as independent variables. In the sixth model (*n* = 65), the dependent variable was happiness during the conflict condition, with PHQ-9 depression score, emotional bank account, and number of family members entered as independent variables. All predictors were entered simultaneously using the Enter method. Only data that showed no evidence of multicollinearity (as indicated by acceptable variance inflation factor and tolerance values) and no significant autocorrelation of residuals (as assessed by Durbin–Watson statistics (Custodio and Sombilla, 2025; Durbin and Watson, 1950, 1951) within the acceptable range) were included in the analyses. Statistical significance was set at *p* < 0.05.

## 3 Results

### 3.1 Participant characteristics and pre-experimental measures

Table 1 summarizes the participant characteristics and age-adjusted pre-experimental measures. Data from nine participants were excluded from HRV analyses (five due to technical errors and four due to excessive ectopic beats); therefore, HRV analyses were conducted on 56 participants, while all 65 participants were included in the overall analyses. Significant sex differences were found in pre-experimental measures. Specifically, women were older than men. To control for this potential confounding effect, analysis of covariance (ANCOVA) was conducted, with age entered as a covariate for all other pre-experimental measures, which are presented as estimated marginal means ± standard error (SE). After adjustment, men exhibited significantly higher Body Mass Index (BMI), Patient Health Questionnaire-9 (PHQ-9) depression scores, and diastolic blood pressure, whereas women reported higher emotional support scores (MOS-SSS subscale). No significant sex differences were observed in pre-experimental systolic blood pressure, heart rate, sleep duration, UCLA trait loneliness scores, Beck anxiety scores, number of family members, or other social support measures (all *p* > 0.05).

**TABLE 1:** Participant characteristics and age-adjusted sex differences in pre-experimental measures (*N* = 65).

Variable	Total (*N* = 65)	Men (*n* = 30)	Women (*n* = 35)	*p*-value	Partial η^2^
Age (years)	48.92 ± 11.69	44.13 ± 11.99	53.03 ± 9.84	0.002	*d* = 0.82
BMI (kg/m^2^)	24.35 ± 3.72	25.88 ± 0.67	23.05 ± 0.61	0.004	0.127
Pre-experiment SBP (mmHg)	139.25 ± 18.25	140.83 ± 3.41	136.03 ± 3.14	0.323	0.016
Pre-experiment DBP (mmHg)	88.74 ± 12.18	92.21 ± 2.24	85.76 ± 2.06	0.046	0.063
Pre-experiment HR (bpm)	76.91 ± 12.27	77.80 ± 2.37	76.15 ± 2.19	0.623	0.004
Years of education	13.82 ± 2.33	13.76 ± 0.45	13.86 ± 0.41	0.877	0.000
Number of family members	3.25 ± 1.29	3.40 ± 0.24	3.12 ± 0.22	0.397	0.012
Number of close relatives	7.38 ± 8.53	7.03 ± 1.56	7.68 ± 1.43	0.768	0.001
Number of close acquaintances	14.43 ± 15.38	15.93 ± 2.97	13.14 ± 2.73	0.508	0.007
Monthly household income	497.23 ± 371.77	516.22 ± 71.72	480.95 ± 66.00	0.729	0.002
Sleep duration (hours)	6.59 ± 1.07	6.57 ± 0.20	6.61 ± 0.18	0.867	0.000
UCLA trait loneliness score	40.15 ± 11.62	43.26 ± 2.17	37.49 ± 2.00	0.064	0.054
PHQ-9 depression score	4.42 ± 4.60	5.86 ± 0.82	3.18 ± 0.75	0.023	0.081
Emotional support score	30.26 ± 7.69	26.73 ± 1.37	33.29 ± 1.26	0.001	0.157
Affectionate support score	11.37 ± 3.71	10.47 ± 0.70	12.14 ± 0.65	0.099	0.043
Beck anxiety score	7.86 ± 6.78	8.68 ± 1.26	7.16 ± 1.16	0.396	0.012

*p*-value, Sex difference; BMI, body mass index; SBP, systolic blood pressure; DBP, diastolic blood pressure; HR, heart rate (beats per minute). Monthly household income is reported in units of 10,000 KRW (approximately USD 6.90 per 10,000 KRW). PHQ-9, Patient Health Questionnaire-9. Emotional support and affectionate support scores were derived from subdomains of the Medical Outcomes Study Social Support Survey (MOS-SSS). Values are presented as mean ± SD for age; all other variables are presented as estimated marginal means ± SE adjusted for age. Cohen’s *d* for age was 0.82, indicating a significant sex difference. As age differed between men and women, all other variables were analyzed using ANCOVA with age as a covariate. Partial η^2^ (partial eta squared) is reported as an effect size measure, with values of 0.01, 0.06, and 0.14 indicating small, medium, and large effects, respectively.

### 3.2 Comparison of emotional, pain, and HRV measures by sex adjusted for age (MANCOVA) (all participants: *N* = 65; HRV subsample: *n* = 56)

Table 2 summarizes the age-adjusted comparisons of emotional, pain, and HRV measures.

**TABLE 2:** Sex differences in emotional, pain, and autonomic responses adjusted for age (MANCOVA) [all participants: *N* = 65 (men = 30, women = 35); HRV subsample: *n* = 56 (men = 30, women = 26)].

Outcome variable	Condition	Men	Women	*F*	*p*	Age × sex interaction (p)	Partial η^2^
Stress	Baseline	44.23 ± 4.71	37.73 ± 4.39	4.525	0.037	0.018	0.069
	Conflict	53.26 ± 4.68	42.82 ± 4.36	3.025	0.087	0.033	0.047
Happiness	Baseline	57.99 ± 3.49	70.43 ± 3.21	6.376	0.014	0.104	0.093
	Conflict	50.20 ± 3.66	64.03 ± 3.36	7.191	0.009	0.021	0.104
Empathy	Baseline	64.63 ± 3.23	71.52 ± 2.97	2.284	*0.136*	0.174	0.036
	Conflict	60.49 ± 2.82	69.21 ± 2.60	4.799	0.032	0.011	0.07
Positive societal perceptions	Baseline	51.74 ± 3.18	65.94 ± 2.93	9.994	0.002	0.586	0.139
	Conflict	43.74 ± 3.06	59.91 ± 2.82	13.985	0.000	0.293	0.184
Negative societal perceptions	Baseline	49.28 ± 3.51	35.76 ± 3.23	7.452	0.008	0.628	0.107
	Conflict	57.90 ± 3.37	45.37 ± 3.10	6.946	0.011	0.538	0.101
Pain	Baseline	10.35 ± 1.18	11.74 ± 1.09	0.691	0.409	0.691	0.011
	Post-conflict	12.62 ± 1.84	20.07 ± 1.70	8.192	0.006	0.438	0.117
lnHF	Baseline	4.62 ± 0.21	5.15 ± 0.23	2.771	0.102	0.754	0.050
	Conflict	4.46 ± 0.21	4.91 ± 0.23	1.808	0.184	0.548	0.033
HF norm	Baseline	46.43 ± 3.92	58.90 ± 4.24	4.219	0.045	0.407	0.074
	Conflict	43.66 ± 3.88	57.71 ± 4.20	5.458	0.023	0.659	0.093
LF norm	Baseline	53.58 ± 3.92	41.10 ± 4.24	4.219	0.045	0.407	0.074
	Conflict	56.34 ± 3.88	42.29 ± 4.20	5.458	0.023	0.659	0.093
LF/HF ratio	Baseline	2.00 ± 0.37	0.94 ± 0.40	3.472	0.068	0.684	0.061
	Conflict	2.14 ± 0.32	0.98 ± 0.34	5.712	0.020	0.936	0.097

lnHF, log-transformed high-frequency power; HF norm, normalized high-frequency HRV scores; LF norm, normalized low-frequency HRV scores. LF-related indices and normalized HRV measures reflect mixed and relative autonomic influences rather than pure sympathetic activity. Values are presented as Madj ± SE (Madj = estimated marginal means adjusted for age; SE = standard error). Partial η^2^ (partial eta squared) = effect size. Positive societal perceptions refer to positive thoughts about society. Negative societal perceptions refer to negative thoughts about society. In the present study, absolute LF power remained stable during conflict, whereas absolute parasympathetic power (lnHF) significantly decreased, indicating selective parasympathetic withdrawal. Accordingly, higher LF norm values should be interpreted as reflecting relative spectral redistribution and autonomic quality rather than direct increases in sympathetic nervous system activity. F, statistic from the analysis of covariance (MANCOVA). *p*, probability value indicating the statistical significance of the observed F statistic. A *p* value < 0.05 indicates statistical significance.

#### 3.2.1 Post-conflict pain (*N* = 65)

**FIGURE 1:**
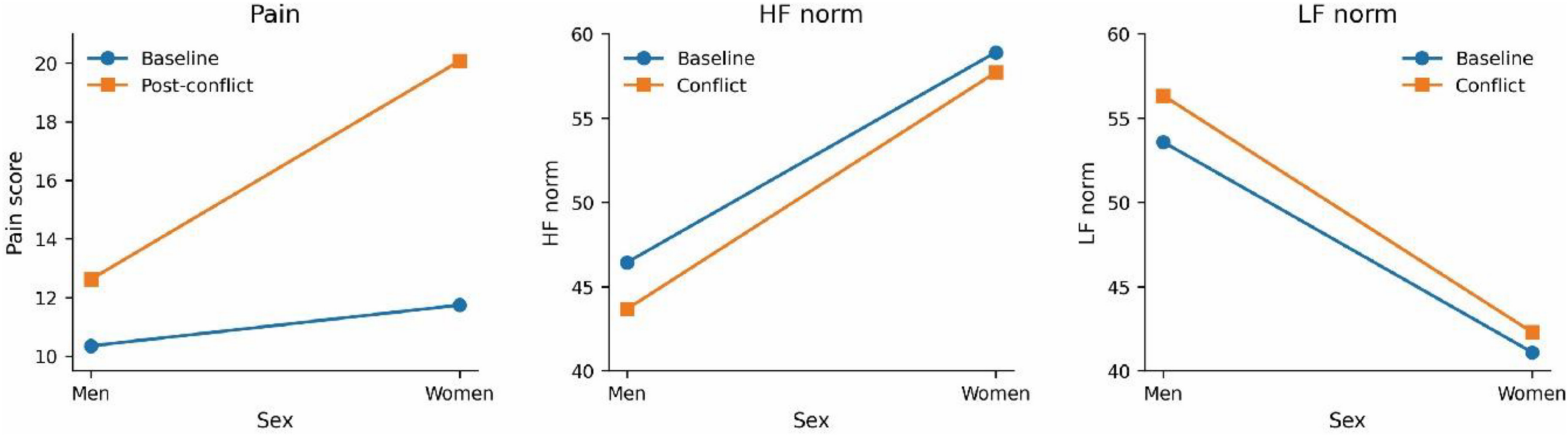
Sex differences in pain and autonomic responses across baseline and conflict conditions. (Left) Pain scores by sex across baseline and post-conflict conditions. No significant sex difference was observed at baseline; however, following the conflict condition, women exhibited a greater increase in pain than men, indicating a context-dependent amplification of pain responses. (Middle) HF norm, an index reflecting relative parasympathetic allocation and autonomic quality, was consistently higher in women than in men at both baseline and during the conflict condition. This pattern indicates greater relative parasympathetic predominance and a more parasympathetically oriented autonomic allocation in women, rather than increased absolute parasympathetic activity. (Right) LF norm was significantly higher in men than in women across both conditions (*p*s < 0.05). However, compared with baseline, absolute LF power (lnLF) remained unchanged during the conflict condition, whereas absolute parasympathetic power (lnHF) significantly decreased (*p* = 0.002), indicating that the principal autonomic response to conflict was selective parasympathetic withdrawal. Accordingly, the higher LF norm in men is best interpreted as greater relative LF predominance secondary to reduced parasympathetic buffering, rather than a genuine increase in sympathetic nervous system activity.

To examine sex differences in pain outcomes while controlling for age, a multivariate analysis of covariance (MANCOVA) was conducted with sex as the between-subjects factor and age as a covariate. The assumption of homogeneity of regression slopes was met, as the sex × age interaction was not significant (*p* = 0.727). Although Box’s M test indicated a violation of the homogeneity of covariance matrices (*p* = 0.027), Pillai’s Trace was employed, as it is widely recommended for MANOVA due to its robustness and is particularly appropriate when this assumption is violated (Nimon, 2012; Olson, 1976) (recent research since 2022 on this issue appears limited). The analysis revealed significant multivariate effects on pain outcomes for both sex (Pillai’s Trace = 0.148, *F*(2, 61) = 5.317, *p* = 0.007) and age (Pillai’s Trace = 0.100, *F*(2, 61) = 3.387, *p* = 0.040). At the univariate level, neither age nor sex showed a significant effect on baseline pain (*p* > 0.05), indicating no sex differences at baseline (Table 2). However, for post-conflict pain measured after the conflict condition, both sex (*F*(1, 62) = 8.192, *p* = 0.006, η^2^ = 0.117) and age (*F*(1, 62) = 4.912, *p* = 0.030, η^2^ = 0.073) were significant predictors. Specifically, women reported significantly higher post-conflict pain than men (Table 2 and Figure 1). Age was negatively associated with post-conflict pain (regression coefficient *B* = –0.248, *p* = 0.030), indicating that higher age was associated with lower post-conflict pain levels across both sexes, as no significant sex × age interaction was observed.

#### 3.2.2 Stress (*N* = 65)

**FIGURE 2:**
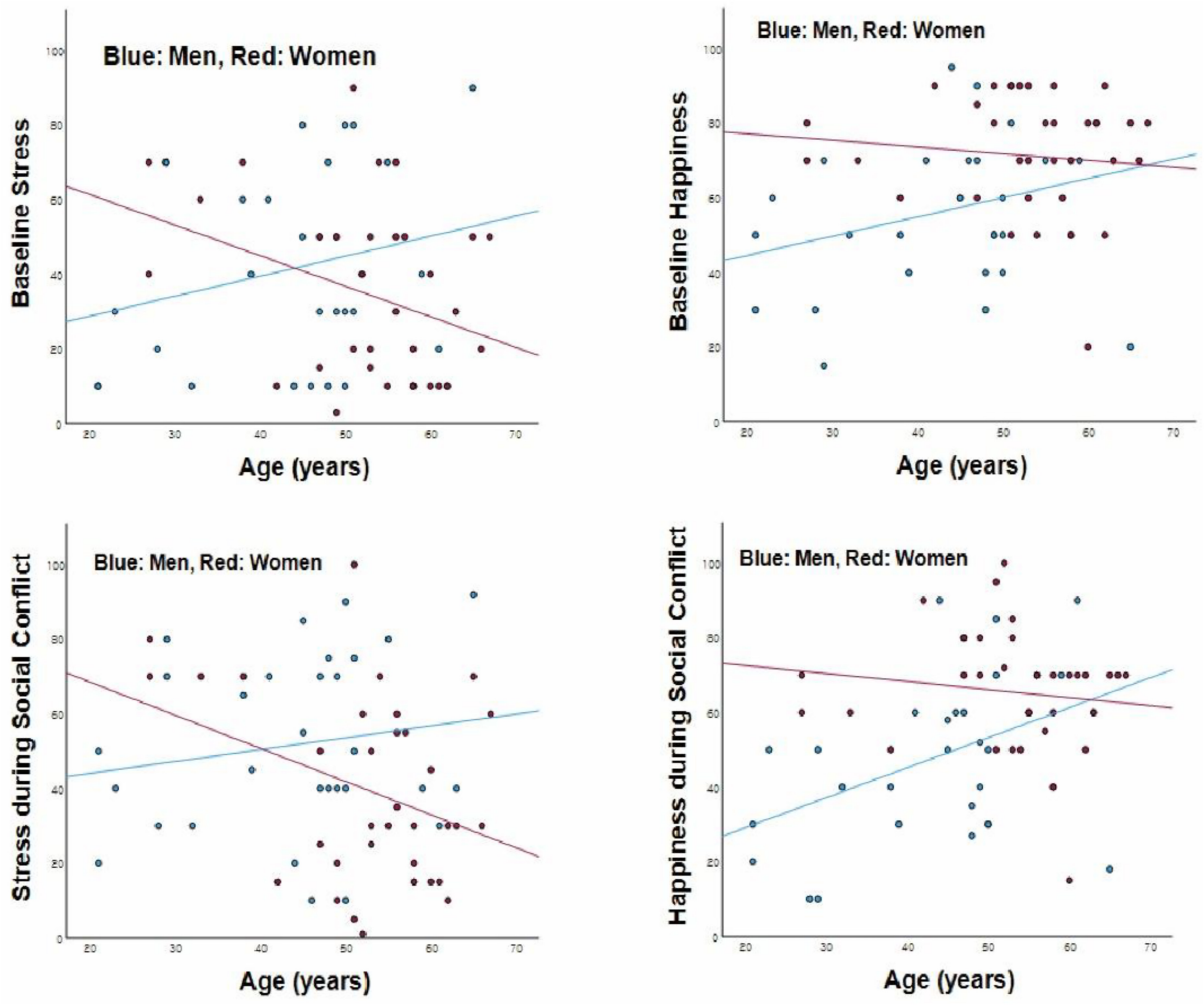
Sex differences in age-related changes in stress and happiness at baseline and during social conflict. Top left: baseline stress. Baseline stress showed opposite age-related patterns by sex. Stress decreased with age in women but showed a slight increase in men, reflecting a significant sex × age interaction. This indicates that age-related changes in baseline stress differ by sex. Bottom left: stress during conflict. Stress during the conflict condition showed a significant sex × age interaction [*F*(1, 61) = 4.746, *p* = 0.033], although the main effects of age and sex were not significant. Stress decreased with age in women but increased with age in men, indicating opposite age-related patterns between men and women. Top right: baseline happiness. In men, baseline happiness showed a slight positive association with age, whereas in women, it showed a weak negative association. Overall, women reported higher baseline happiness than men across the age range. Bottom right: happiness during conflict. In men, happiness during conflict increased with age, whereas in women, it showed a negative association with age. This opposing pattern reflects the significant sex × age interaction. Women generally reported higher happiness levels than men across most ages, although the difference narrowed with increasing age.

The multivariate analysis revealed no significant main effects of sex [Pillai’s Trace = 0.070, *F*(2, 60) = 2.270, *p* = 0.112] or age [Pillai’s Trace = 0.029, *F*(2, 60) = 0.885, *p* = 0.418] on stress outcomes. The interaction between sex and age showed a marginal trend at the multivariate level [Pillai’s Trace = 0.088, *F*(2, 60) = 2.910, *p* = 0.062]. At the univariate level, for baseline stress, a significant interaction between sex and age was observed [*F*(1, 61) = 5.914, *p* = 0.018, η^2^ = 0.088], along with a significant main effect of sex [*F*(1, 61) = 4.525, *p* = 0.037, η^2^ = 0.069], while age was not significant (*p* = 0.613). For stress during the conflict condition, a significant sex × age interaction was also found [*F*(1, 61) = 4.746, *p* = 0.033, η^2^ = 0.072], whereas neither the main effect of sex (*p* = 0.087) nor age (*p* = 0.307) reached statistical significance. Estimated marginal means indicated that men showed higher stress levels than women at both baseline (Men: *M* = 44.23, Women: *M* = 37.73) and during the conflict condition (Men: *M* = 53.26, Women: *M* = 42.82). The reduction in stress observed in women during the conflict condition attenuated the baseline sex difference, such that the initially significant main effect of sex became non-significant under conflict (Table 2). Follow-up regression analyses conducted separately for men and women revealed a divergent pattern in the relationship between age and stress across sexes. In women, age was negatively associated with stress both at baseline (*B* = –0.819, *p* = 0.054) and in the conflict condition (*B* = –0.888, *p* = 0.036), indicating a tendency for stress to decrease with increasing age, with the effect reaching statistical significance in the conflict condition. In contrast, in men, age was positively associated with stress, with estimated effects of *B* = 0.536 at baseline and B = 0.318 in the conflict condition, indicating that stress tended to increase with age. Notably, the interaction between sex and age was significant at baseline (*B* = 1.355, *p* = 0.018) and in the conflict condition (*B* = 1.206, *p* = 0.033), indicating that the effect of age on stress differed significantly by sex, with stress decreasing with age in women but increasing with age in men (Figure 2).

#### 3.2.3 Happiness (*N* = 65)

The multivariate test revealed a significant main effect of sex [Pillai’s Trace = 0.129, *F*(2, 60) = 4.435, *p* = 0.016], indicating overall differences between men and women across conditions. In contrast, the main effect of age was not significant [Pillai’s Trace = 0.037, *F*(2, 60) = 1.156, *p* = 0.322], suggesting that age did not significantly influence happiness levels. The interaction between sex and age approached significance [Pillai’s Trace = 0.091, *F*(2, 60) = 3.002, *p* = 0.057], indicating a potential moderating role of age. Univariate analyses showed that sex significantly affected baseline happiness [*F*(1, 62) = 6.376, *p* = 0.014, η^2^ = 0.093] and happiness in the conflict condition [*F*(1, 62) = 7.191, *p* = 0.009, η^2^ = 0.104], with women reporting higher happiness levels than men in both conditions (Table 2). Age did not significantly predict happiness in either condition (baseline: *p* = 0.328; conflict: *p* = 0.115). Importantly, a significant interaction between sex and age was observed for happiness during the conflict condition [*F*(1, 61) = 5.618, *p* = 0.021], indicating that the relationship between age and happiness differed by sex during the conflict condition. Descriptive statistics indicated that happiness decreased from baseline to the conflict condition in both groups; however, women maintained consistently higher happiness levels than men across both conditions. Follow-up regression analyses further clarified the nature of the sex × age interaction in happiness. In women, age was negatively associated with happiness both at baseline (*B* = -0.180, *p* = 0.570) and in the conflict condition (*B* = -0.216, *p* = 0.504), indicating a tendency for happiness to decrease with increasing age, although these effects were not statistically significant. In contrast, in men, age was positively associated with happiness, with estimated effects of *B* = 0.515 at baseline and *B* = 0.805 in the conflict condition (Figure 2), indicating that happiness tended to increase with age. Notably, the interaction between sex and age was significant in the conflict condition (*B* = 1.021, *p* = 0.021), indicating that the effect of age on happiness differed significantly by sex in the conflict condition, with happiness decreasing with age in women but increasing with age in men.

#### 3.2.4 Empathy (*N* = 65)

The multivariate analysis revealed a significant main effect of sex [Pillai’s Trace = 0.140, *F*(2, 60) = 4.886, *p* = 0.011], indicating overall differences between men and women in empathy across conditions. The main effect of age was not significant (*p* = 0.476), whereas the interaction between sex and age was significant [Pillai’s Trace = 0.107, *F*(2, 60) = 3.603, *p* = 0.033], suggesting that the relationship between age and empathy differed by sex. For empathy at baseline, age was not significantly associated with empathy in women (*B* = −0.031, *p* = 0.916). In men, the estimated effect of age was positive (*B* = 0.508), although the sex × age interaction was not statistically significant (*B* = 0.539, *p* = 0.174), indicating a non-significant tendency for empathy to increase with age in men but remain stable in women. In the conflict condition, age showed a non-significant negative association with empathy in women (*B* = -0.320, *p* = 0.199), whereas in men, age was positively associated with empathy (*B* = 0.547). Notably, the sex × age interaction was significant (*B* = 0.867, *p* = 0.011), indicating that the effect of age on empathy differed by sex, with empathy decreasing with age in women but increasing in men. Adjusted means indicated that women reported higher empathy than men, particularly in the conflict condition (*p* = 0.032), whereas the difference at baseline was not statistically significant (Table 2).

#### 3.2.5 Positive and negative societal thoughts (*N* = 65)

Women reported significantly higher positive societal thoughts than men at both baseline (*B* = -14.202, *p* = 0.002) and during the conflict condition (*B* = -16.171, *p* < 0.001). Conversely, men reported higher negative societal thoughts than women at baseline (*B* = 13.526, *p* = 0.008) and during the conflict condition (*B* = 12.527, *p* = 0.011). Age was not a significant predictor for either variable (Table 2).

#### 3.2.6 HF norm-HRV (*n* = 56)

The multivariate effect of sex approached significance [Pillai’s Trace = 0.105, *F*(2, 52) = 3.051, *p* = 0.056, η^2^ = 0.105], whereas age did not show a significant multivariate effect [Pillai’s Trace = 0.001, *F*(2, 52) = 0.029, *p* = 0.971, η^2^ = 0.001]. At the univariate level, sex showed significant effects on HF norm at both baseline [*F*(1, 53) = 4.219, *p* = 0.045, η^2^ = 0.074] and during the conflict condition [*F*(1, 53) = 5.458, *p* = 0.023, η^2^ = 0.093], with women exhibiting higher HF norm than men in both conditions (Figure 1). Age was not a significant predictor of HF norm at either baseline (*p* = 0.813) or during the conflict condition (*p* = 0.928). Pairwise comparisons further confirmed that women had significantly higher HF norm than men at baseline (mean difference = 12.47, *p* = 0.045) and during the conflict condition (mean difference = 14.05, *p* = 0.023) (Table 2). Because HF norm reflects the relative allocation of autonomic resources rather than absolute parasympathetic activity, these findings suggest that women maintained greater relative parasympathetic predominance and higher autonomic quality across both conditions.

#### 3.2.7 LF norm-HRV (*n* = 56)

The multivariate effect of sex approached significance [Pillai’s Trace = 0.105, *F*(2, 52) = 3.051, *p* = 0.056, η^2^ = 0.105], whereas age was not significant [Pillai’s Trace = 0.001, *F*(2, 52) = 0.029, *p* = 0.971, η^2^ = 0.001]. Because normalized spectral measures reflect proportional allocation rather than absolute autonomic power, the univariate analyses of LF norm mirrored the findings for HF norm. Specifically, sex showed significant effects on LF norm at both baseline [*F*(1, 53) = 4.219, *p* = 0.045, η^2^ = 0.074] and during the conflict condition [*F*(1, 53) = 5.458, *p* = 0.023, η^2^ = 0.093], with men exhibiting significantly higher LF norm than women in both conditions (Figure 1). Age was not a significant predictor of LF norm in either condition. Estimated marginal means adjusted for age further indicated that men maintained higher LF norm than women at baseline (53.58 ± 3.92 vs. 41.10 ± 4.24, *p* = 0.045) and during the conflict condition (56.34 ± 3.88 vs. 42.29 ± 4.20, *p* = 0.023) (Table 2). Because LF norm reflects relative spectral composition rather than absolute LF power, the higher LF norm observed in men should be interpreted as greater relative LF predominance resulting from autonomic spectral redistribution secondary to reduced parasympathetic buffering, rather than as evidence of increased sympathetic activation.

#### 3.2.8 LF/HF ratio (*n* = 56)

The multivariate analysis revealed no significant main effect of age [Pillai’s Trace = 0.001, *F*(2, 52) = 0.026, *p* = 0.974] and no significant sex × age interaction [Pillai’s Trace = 0.007, *F*(2, 51) = 0.179, *p* = 0.837], indicating that age did not influence LF/HF ratios or their relationship with sex. The multivariate main effect of sex approached significance [Pillai’s Trace = 0.100, *F*(2, 52) = 2.900, *p* = 0.064, η^2^ = 0.100]. At the univariate level, sex showed a significant effect on LF/HF ratio during the conflict condition [*F*(1, 53) = 5.712, *p* = 0.020, η^2^ = 0.097], with men exhibiting higher values than women. In contrast, the baseline difference did not reach statistical significance [*F*(1, 53) = 3.472, *p* = 0.068], although a trend in the same direction was observed. Estimated marginal means further indicated that men showed higher LF/HF ratios than women at baseline (2.00 ± 0.37 vs. 0.94 ± 0.40, *p* = 0.068) and during the conflict condition (2.14 ± 0.32 vs. 0.98 ± 0.34, *p* = 0.020) (Table 2). Because the LF/HF ratio reflects relative spectral composition rather than absolute autonomic activity, and because absolute LF power remained stable whereas absolute parasympathetic power (lnHF) significantly decreased during the conflict condition, the elevated LF/HF ratio observed in men is best interpreted as greater relative LF predominance resulting from autonomic spectral redistribution secondary to reduced parasympathetic buffering, rather than increased sympathetic activation.

#### 3.2.9 lnHF (log-transformed high-frequency power of HRV; *n* = 56)

In contrast to the normalized spectral measures, no significant main effects or interactions involving sex were observed for absolute lnHF power (Table 2). However, lnHF showed a significant negative association with age at baseline (*B* = -0.033, *p* = 0.019), indicating an age-related decline in absolute parasympathetic capacity and overall autonomic reserve. A similar negative trend was observed during the conflict condition but did not reach statistical significance.

Thus, the observed sex differences appear to reflect differences in relative spectral composition and autonomic quality rather than differences in absolute parasympathetic power. These findings indicate that sex differences were evident primarily in the relative distribution of autonomic resources (autonomic quality) rather than in absolute parasympathetic power (autonomic quantity). In other words, although men and women did not substantially differ in absolute parasympathetic power, they differed in how autonomic resources were proportionally allocated and balanced across conditions.

### 3.3 Regression analysis of all 65 participants

**FIGURE 3:**
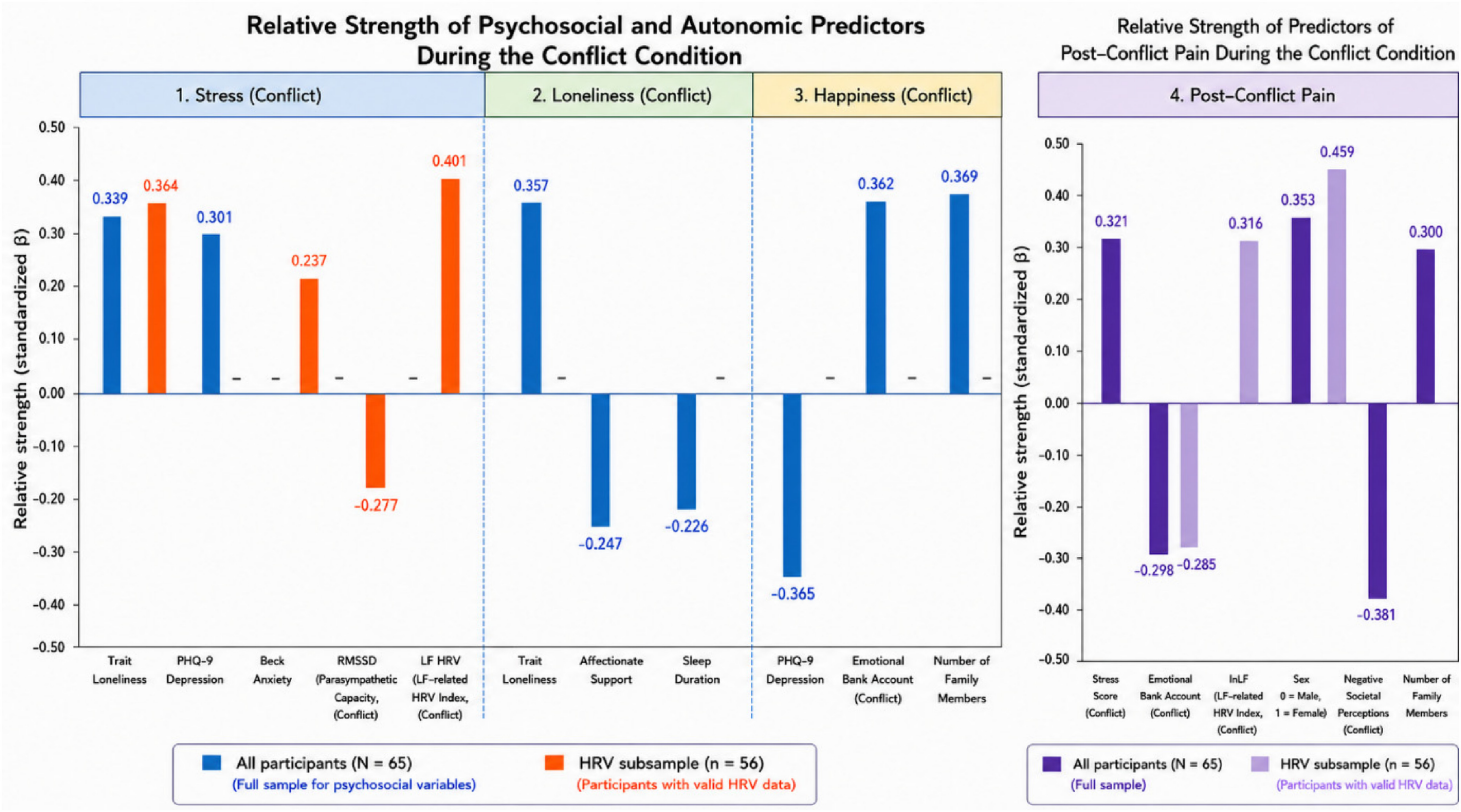
Relative strength of psychosocial and autonomic predictors of stress, loneliness, happiness, and post-conflict pain. Bars represent standardized regression coefficients (β) derived from the multiple regression models summarized in Table 3. Positive coefficients indicate predictors associated with higher outcome values, whereas negative coefficients indicate predictors associated with lower outcome values. Stress, loneliness, and happiness models were derived from variables measured during the conflict condition, whereas the pain model represents predictors of post-conflict pain assessed immediately after the conflict condition. In the stress panel, blue bars represent the full sample (*N* = 65) and orange bars represent the HRV subsample with valid autonomic recordings (*n* = 56). In the post-conflict pain panel, dark purple bars represent the full sample (*N* = 65) and light purple bars represent the HRV subsample (*n* = 56). Predictors displayed with a single colored bar were included only in the corresponding regression model. A dash (–) indicates that the predictor was not included in the corresponding regression model. HRV, heart rate variability; RMSSD, root mean square of successive R-R interval differences, reflecting parasympathetic regulatory capacity; LF HRV, low-frequency HRV indices reflecting mixed sympathetic and parasympathetic influences rather than pure sympathetic activity; lnLF, natural log-transformed low-frequency HRV. Standardized regression coefficients (β) allow comparison of the relative strength of predictors independent of their original measurement scales. Because absolute LF power remained stable while absolute parasympathetic power (lnHF) decreased during the conflict condition, LF-related indices should be interpreted as reflecting relative spectral redistribution (autonomic quality) rather than direct increases in sympathetic nervous system activity.

The standardized regression coefficients (β) are summarized in Table 3 and Figure 3.

**TABLE 3:** Integrated regression results identifying predictors of psychological outcomes during the conflict condition.

Dependent variable	Significant predictor	Standardized β (*N* = 65/*n* = 56)	Direction of effect	Interpretation
Stress (Conflict)	Trait loneliness	0.339/0.364	Positive (+)	Higher trait loneliness → higher stress
	PHQ-9 depression	0.301/–	Positive (+)	Higher depression → higher stress
	Beck anxiety	–/0.237	Positive (+)	Higher chronic anxiety → higher stress
	RMSSD (parasympathetic capacity, conflict)	–/-0.277	Negative (–)	Greater parasympathetic regulation → lower stress
	LF HRV (LF-related HRV Index, Conflict)	–/0.401	Positive (+)	Relative LF predominance → higher stress
Post-conflict pain	Stress score (Conflict)	0.321/–	Positive (+)	Higher stress → more pain
	Emotional bank account (Conflict)	-0.298/-0.285	Negative (–)	Stronger emotional resources → less pain
	lnLF (LF-related HRV Index, Conflict)	–/0.316	Positive (+)	Relative LF predominance → greater pain
	Sex (0 = Male, 1 = Female)	0.353/0.459	Positive (+)	Women reported higher pain than men
	Negative societal perceptions (Conflict)	-0.381/–	Negative (–)	More negative social thoughts → less pain
	Number of family members	0.300/–	Positive (+)	More family members → more pain
Loneliness (Conflict)	Trait loneliness	0.357/–	Positive (+)	Higher trait loneliness → higher loneliness
	Affectionate support	-0.247/–	Negative (–)	Greater emotional support → lower loneliness
	Sleep duration	-0.226/–	Negative (–)	Longer sleep → lower loneliness
Happiness (Conflict)	PHQ-9 depression	-0.365/–	Negative (–)	Higher depression → lower happiness
	Emotional bank account (Conflict)	0.362/–	Positive (+)	Greater emotional resources → higher happiness
	Number of family members	0.369/–	Positive (+)	More family members → higher happiness

HRV, heart rate variability; RMSSD, root mean square of successive R–R interval differences; LF HRV, low-frequency HRV indices reflecting mixed sympathetic and parasympathetic influences rather than pure sympathetic activity; lnLF = log-transformed low-frequency HRV. Conflict = conflict condition. Negative societal perceptions refer to negative thoughts about society. In the present study, absolute LF power remained stable during conflict, whereas absolute parasympathetic power (lnHF) significantly decreased, indicating selective parasympathetic withdrawal. Accordingly, associations involving LF-related indices should be interpreted as reflecting relative spectral redistribution and autonomic quality rather than direct increases in sympathetic nervous system activity. Where two β values are provided (e.g., 0.339/0.364), the first is based on the full sample (*N* = 65; section 3.5), and the second is based on the HRV subsample (*n* = 56; section 3.6). The HRV subsample included only participants with valid HRV recordings. Data from five participants were excluded due to technical errors during HRV measurement, and four additional participants were excluded due to excessive ectopic beats (>2 per minute). These participants were retained in the full sample (*N* = 65) for analyses not involving HRV. A dash (–) indicates that the predictor was not included in the respective regression model. All β values represent standardized regression coefficients, and only statistically significant predictors are reported. All analyses pertain to the conflict image viewing condition. Standardized coefficients (β) allow comparison of the relative strength of predictors independent of their original measurement scales.

#### 3.3.1 Stress (conflict condition) = 8.501 + 0.728 × (UCLA trait loneliness) + 1.639 × (PHQ-9 depression score)

When viewing social conflict images, the regression equation predicting stress during the conflict condition in the full sample (*N* = 65) was as follows: *Stress (conflict condition) = 8.501 + 0.728 × (UCLA trait loneliness) + 1.639 × (depression score).* The overall model was statistically significant (*R*^2^ = 0.310, adjusted *R*^2^ = 0.288, *F* = 13.932, *p* < 0.001). Both UCLA trait loneliness and depression were significant positive predictors of stress during the conflict condition (*p* = 0.008 and *p* = 0.017, respectively).

#### 3.3.2 Post-conflict pain (Y) = 19.752 + 7.182 × (sex [0 = male, 1 = female]) + 2.384 × (number of family members) – 0.166 × (emotional bank account during conflict) + 0.131 × (stress during conflict) – 0.210 × (negative societal thoughts during conflict)

The regression equation predicting pain after the conflict condition was as follows: *Post-conflict pain (Y) = 19.752 + 7.182 × (sex [0 = male, 1 = female]) + 2.384 × (number of family members) - 0.166 × (emotional bank account during conflict) + 0.131 × (stress during conflict) - 0.210 × (negative societal thoughts during conflict).* The overall model was statistically significant (*R*^2^ = 0.334, adjusted *R*^2^ = 0.278, *F* = 5.917, *p* < 0.001). Sex (coded 0 = male, 1 = female), number of family members, and stress during the conflict condition were significant positive predictors of post-conflict pain (*p* = 0.004, *p* = 0.009, and *p* = 0.011, respectively). Because sex was coded with males as the reference group, the positive coefficient indicates that female participants reported higher pain levels than males. In contrast, emotional bank account and negative societal thoughts during the conflict condition were significant negative predictors of post-conflict pain (*p* = 0.013 and *p* = 0.004, respectively).

#### 3.3.3 Loneliness (conflict condition) = 54.35 + 0.71 × (UCLA trait loneliness) - 1.54 × (affectionate support) - 4.85 × (sleep duration)

When viewing social conflict images, the regression equation predicting loneliness during the conflict condition was as follows: Loneliness (conflict condition) = 54.35 + 0.71 × (UCLA trait loneliness) - 1.54 × (affectionate support) - 4.85 × (sleep duration).The overall model was statistically significant (*R*^2^ = 0.323, adjusted *R*^2^ = 0.290, *F* = 9.702, *p* < 0.001). Pre-experimental trait loneliness was a significant positive predictor of loneliness during the conflict condition (*p* = 0.005), whereas affectionate support and sleep duration (hours) were significant negative predictors (*p* = 0.050 and *p* = 0.036, respectively). The Affectionate Support score is a subdomain of the MOS-SSS, comprising three items that assess perceived emotional support and affectionate relationships.

#### 3.3.4 Happiness (conflict condition) = 20.56 - 0.67 × (PHQ-9 depression score) + 5.93 × (number of family members) + 0.45 × (emotional bank account during conflict)

The regression equation predicting happiness during the conflict condition was as follows: *Happiness (conflict condition) = 20.56 - 0.67 × (PHQ-9 depression score) + 5.93 × (number of family members) + 0.45 × (emotional bank account during conflict).* The overall model was statistically significant *(R*^2^ = 0.416, adjusted *R*^2^ = 0.388, *F* = 14.501, *p* < 0.001). Pre-experimental depression (assessed using PHQ-9) was a significant negative predictor of happiness during the conflict condition (*p* < 0.001), whereas emotional bank account during the conflict condition and number of family members were significant positive predictors (*p* < 0.001 for both). The emotional bank account was operationalized as a state-like measure reflecting participants’ perceived emotional investment or replenishment during the conflict condition—analogous to “making a deposit.”

### 3.4 Regression analysis of 56 participants with valid HRV data

The standardized regression coefficients (β) are summarized in Table 3 and Figure 3.

#### 3.4.1 Stress (conflict condition) = 11.707 - 0.459 × (RMSSD during conflict) + 0.043 × (LF-HRV during conflict) + 0.737 × (UCLA trait loneliness) + 0.858 × (Beck anxiety score)

Stress during the conflict condition was analyzed in the subsample with valid HRV data (*n* = 56). A multiple regression analysis was conducted to identify predictors of stress. *Stress (conflict condition) = 11.707 - 0.459 × (RMSSD during conflict) + 0.043 × (LF-HRV during conflict) + 0.737 × (UCLA trait loneliness) + 0.858 × (Beck anxiety score).* The overall model was statistically significant (*R*^2^ = 0.430, adjusted *R*^2^ = 0.385, *F* = 9.620, *p* < 0.001). LF-HRV during the conflict condition, pre-experimental UCLA trait loneliness, and Beck anxiety were significant positive predictors of stress (*p* = 0.003, *p* = 0.037, and *p* = 0.002, respectively). In contrast, RMSSD during the conflict condition was a significant negative predictor (*p* = 0.035).

#### 3.4.2 Post-conflict pain (Y) = 6.772 + 9.438 × (sex [0 = male, 1 = female]) + 3.201 × (lnLF during conflict) - 0.160 × (emotional bank account during conflict)

The regression equation predicting post-conflict pain in participants with valid HRV data (*n* = 56) was as follows: Post-conflict pain (Y) = 6.772 + 9.438 × (sex [0 = male, 1 = female]) + 3.201 × (lnLF during conflict) – 0.160 × (emotional bank account during conflict). The overall model was statistically significant (*R*^2^ = 0.266, adjusted *R*^2^ = 0.223, *F* = 6.266, *p* = 0.001). Sex (coded 0 = male, 1 = female) and lnLF during the conflict condition were significant positive predictors of post-conflict pain (*p* = 0.001 and *p* = 0.013, respectively), whereas emotional bank account during the conflict condition was a significant negative predictor (*p* = 0.024). Because sex was coded with males as the reference group, the positive coefficient indicates that female participants reported higher pain levels than males.

### 3.5 Differences in self-reported emotional responses between baseline and conflict conditions (*N* = 65)

All self-reported emotional variables differed significantly between the baseline and conflict conditions (all *ps* ≤ 0.041; Table 4). Compared to baseline, the conflict condition was associated with higher levels of stress, negative thoughts about society, anxiety, fear, and state loneliness, as well as increased pain. In contrast, happiness, empathy, positive thoughts about society, and emotional bank account were significantly lower in the conflict condition. Overall, these findings indicate that exposure to conflict stimuli induces increased psychological distress and pain, alongside reduced positive emotional and social functioning.

**TABLE 4:** Self-reported emotional differences between the baseline and conflict conditions (all participants: *N* = 65).

Measure	Baseline	Conflict	*p*	Cohen’s *d*
Stress	37.74 ± 24.71	44.97 ± 24.99	<0.001	–0.619
Happiness	64.69 ± 19.50	57.65 ± 21.05	<0.001	0.698
Empathy	68.34 ± 17.56	65.18 ± 15.53	0.041	0.259
Positive thoughts about society	59.38 ± 18.17	52.45 ± 17.79	<0.001	0.722
Negative thoughts about society	42.00 ± 19.36	51.15 ± 18.51	<0.001	–1.029
Emotional bank account	65.92 ± 17.68	59.35 ± 18.31	<0.001	0.838
Anxiety	30.11 ± 21.64	37.46 ± 21.47	<0.001	–0.637
Fear	24.28 ± 21.30	33.00 ± 22.09	<0.001	–0.860
State loneliness	27.45 ± 24.33	33.31 ± 23.07	<0.001	–0.668
Pain	11.10 ± 6.14	16.63 ± 10.22	<0.001	–0.737

Values are presented as mean ± standard deviation (SD). A negative Cohen’s *d* indicates that the mean in the conflict condition is higher than that in the baseline condition. Pain at baseline refers to post-baseline pain measured after the baseline condition, whereas pain in the conflict condition refers to post-conflict pain measured after the conflict condition.

### 3.6 Differences in autonomic nervous system responses between baseline and conflict conditions: comparison of HRV data from 56 participants with valid measurements

Among the 56 participants with valid HRV data, significant condition effects were observed primarily in indices reflecting absolute parasympathetic activity (Table 5). Specifically, lnHF was significantly lower in the conflict condition than at baseline, indicating a marked reduction in parasympathetic (vagal) activity during conflict exposure. Consistent with this finding, absolute HF power was also significantly reduced during the conflict condition. In contrast, neither LF power nor lnLF significantly differed between baseline and conflict conditions. Taken together, these findings indicate that exposure to conflict stimuli primarily induced selective parasympathetic withdrawal rather than alterations in absolute LF activity. Because absolute LF power remained stable whereas absolute parasympathetic power (lnHF) significantly decreased, the principal autonomic response to conflict appears to reflect selective parasympathetic withdrawal. Thus, exposure to conflict stimuli primarily reduced absolute parasympathetic activity without evidence of changes in absolute LF activity. Accordingly, any changes observed in normalized HRV measures should be interpreted within the context of relative spectral redistribution and autonomic quality rather than as evidence of absolute autonomic activation or direct changes in sympathetic nervous system activity. Heart rate was higher at baseline than during the conflict condition (Table 5). This unexpected finding contrasts with the typical stress-related increase in heart rate and may reflect parasympathetically mediated cardiac deceleration, which has been reported during passive viewing of threatening or emotionally salient stimuli (Bradley et al., 2001).

**TABLE 5:** Differences in autonomic nervous system responses between baseline and conflict conditions (HRV subsample: *n* = 56).

Measure	Baseline	Conflict	*p*	Cohen’s *d*
HF	248.76 ± 379.51	208.13 ± 315.52	0.015	0.334
lnHF	4.87 ± 1.12	4.67 ± 1.12	0.002	0.439
RMSSD	24.27 ± 16.74	23.03 ± 14.94	0.064	0.252
LF	194.17 ± 237.42	181.28 ± 230.10	0.661	0.059
lnLF	4.76 ± 1.04	4.66 ± 1.02	0.374	0.120
Heart rate	75.77 ± 11.48	74.68 ± 11.03	0.001	0.469
HF norm	52.22 ± 21.03	50.18 ± 21.04	0.412	0.111
LF norm	47.27 ± 21.00	50.53 ± 20.60	0.167	-0.187
LF/HF ratio	1.51 ± 1.96	1.60 ± 1.72	0.644	-0.062

Values are presented as mean ± standard deviation (SD). The high-frequency (HF) component of heart rate variability (HRV) primarily reflects parasympathetic (vagal) activity. lnHF = log-transformed high-frequency power. The low-frequency (LF) component of HRV reflects mixed sympathetic and parasympathetic influences rather than pure sympathetic activity. lnLF = log-transformed low-frequency power. RMSSD = root mean square of successive differences between adjacent R–R intervals. Heart rate (HR) = beats per minute. HF norm = normalized high-frequency HRV scores. LF norm = normalized low-frequency HRV scores.

Importantly, the present study distinguishes between autonomic quantity (absolute spectral power; e.g., HF and LF power) and autonomic quality, defined as the relative allocation of autonomic resources and represented by relative spectral measures such as the LF/HF ratio, HF norm, and LF norm. Because LF-related and normalized HRV indices reflect mixed and relative autonomic influences, they should not be interpreted as direct measures of sympathetic activation or withdrawal. Rather, these indices should be interpreted as indicators of the relative allocation and balance of autonomic resources within the overall autonomic space. Accordingly, the present findings are best understood as reflecting selective parasympathetic withdrawal and relative spectral redistribution, rather than a pure increase or decrease in sympathetic nervous system activity.

## 4 Discussion

Exposure to conflict-related stimuli elicited a coordinated psychophysiological response characterized by selective parasympathetic withdrawal and a relative shift toward sympathetic predominance rather than an active sympathetic surge. These autonomic changes were accompanied by heightened psychological vulnerability, elevated negative affect, diminished prosocial emotional functioning, and increased pain. Age-adjusted analyses revealed distinct sex differences, with women showing greater parasympathetic dominance and more adaptive emotional responses, yet higher post-conflict pain, whereas men exhibited a relative shift toward sympathetic predominance resulting from reduced parasympathetic activity rather than an absolute increase in sympathetic activity, as reflected by higher normalized LF-HRV values. Significant sex-by-age interactions indicated divergent age-related trajectories in stress, happiness, and empathy. Furthermore, regression analyses demonstrated that stress and pain were associated with autonomic imbalance and psychological factors, including loneliness, depression, and social cognition, highlighting integrated mechanisms underlying conflict-induced distress.

The present findings provide important insight into the multifactorial determinants of pain following exposure to social conflict, highlighting the integrated roles of biological, psychological, and social factors. Notably, post-conflict pain was significantly higher in women than in men, and female sex emerged as the strongest predictor of pain across all regression models, exceeding the contributions of autonomic, psychological, and social variables. This finding is consistent with extensive literature demonstrating that women typically exhibit lower pain thresholds and greater pain sensitivity than men (Choi et al., 2016; Karos et al., 2024; Keogh, 2021), suggesting that sex-related biological and neurophysiological mechanisms play a central role in pain amplification under socially stressful conditions. Importantly, these differences were not observed at baseline, indicating that sex-specific disparities in pain are particularly pronounced under conditions of social conflict rather than reflecting general baseline differences. In addition to sex, autonomic and psychological factors significantly contributed to pain outcomes. Importantly, the association between lnLF-HRV and post-conflict pain should not be interpreted as evidence of a pure sympathetic activation effect. Because the LF band reflects a composite signal influenced by both sympathetic and parasympathetic inputs and absolute LF power did not significantly increase during conflict exposure, the observed association is more appropriately interpreted as reflecting a relative shift toward sympathetic predominance arising from parasympathetic withdrawal and autonomic imbalance. Similarly, stress levels during the conflict condition were positively associated with pain, suggesting that emotional distress and reductions in parasympathetic buffering jointly contribute to heightened pain perception and nociceptive processing.

Social-contextual factors also played a significant role in shaping both psychological and pain responses. Integrated regression analyses revealed a dual-edged role of family size across emotional and somatic outcomes. Each additional family member was associated with an increase of 5.93 points in happiness and 2.384 points in post-conflict pain (β = 0.369 and 0.300, respectively). Because both outcomes were measured using the same 0–100 Numerical Rating Scale and both regression models showed robust explanatory power (Happiness: *R*^2^ = 0.416; Post-conflict pain: *R*^2^ = 0.334; both *p* < 0.001), the standardized coefficients suggest a somewhat stronger association with happiness than with pain, indicating that the emotional benefits of a larger family network may slightly outweigh its somatic costs. This pattern is consistent with a trade-off between social support and social burden. Previous research has shown that social support—particularly emotional support—plays a crucial role in alleviating chronic pain (Gong et al., 2024), indicating that the mere presence of more family members does not necessarily guarantee beneficial effects. Accordingly, larger family networks may simultaneously provide emotional resources while increasing interpersonal responsibilities and emotional demands. Because post-conflict pain was assessed immediately after the conflict condition using standardized von Frey stimulation, the observed positive association between family size and pain likely reflects stress-induced somatic hypersensitivity. Overall, these findings suggest that larger family networks may increase vulnerability to stress-related pain, yet their overall contribution to psychological well-being appears to be more substantial. In contrast, emotional resources appeared to exert a consistently protective effect. The emotional bank account—a state-like construct reflecting perceived emotional investment and relational security during the conflict condition—was positively associated with happiness while simultaneously predicting lower post-conflict pain, indicating that individuals with greater emotional resilience and relational security experienced both enhanced psychological well-being and attenuated pain responses. These findings support the buffering role of emotional support and internal psychological resources in mitigating stress-related physiological reactivity and suggest that, compared with structural aspects of social relationships, including family size, the perceived quality of emotional relationships may constitute a more consistently protective factor for both well-being and pain regulation.

Beyond the protective role of emotional resources, another intriguing finding was the negative association between negative thoughts about society and post-conflict pain. Although counterintuitive, this result can be interpreted through the framework of self-verification theory (Ayduk et al., 2013), which posits that individuals experience reduced physiological stress when external experiences align with their pre-existing beliefs. This interpretation is further supported by recent work on self-congruency, suggesting that alignment between external experiences and one’s internal self-concept enhances perceived authenticity and reduces internal conflict, particularly when such experiences are cognitively salient and perceived as self-relevant (Huber et al., 2025). In the present study, individuals holding more negative views of society may have perceived conflict as expected and consistent with their worldview, thereby attenuating physiological reactivity, including pain responses. This apparent dissociation between subjective negativity and reduced pain highlights the important role of cognitive appraisal in modulating stress-related physiological responses. Taken together, these findings suggest that post-conflict pain is not determined solely by physiological activation but emerges from a dynamic interplay among autonomic regulation, social-contextual influences, emotional resources, and cognitive appraisal processes. This integrative perspective underscores the importance of addressing both physiological regulation and psychosocial factors when developing interventions to reduce pain in socially stressful contexts.

Participants exposed to conflict imagery exhibited increased psychological distress, including higher state loneliness, anxiety, stress, and pain, alongside a marked withdrawal of parasympathetic regulation (reduced lnHF-HRV), emotional resources, positive social cognitions, empathy, and happiness. These findings indicate that social conflict stimuli effectively disrupt both affective and physiological regulation, consistent with prior research linking interpersonal conflict to heightened distress and impaired relational functioning (Vallone and Zurlo, 2024). UCLA trait loneliness emerged as a significant predictor of stress, underscoring the role of social disconnection in amplifying emotional responses. Additionally, state loneliness during the conflict condition was positively predicted by trait loneliness and negatively predicted by affectionate support and sleep duration (*R*^2^ = 0.323, adjusted *R*^2^ = 0.290, *F* = 9.702, *p* < 0.001). These findings suggest that trait loneliness increases vulnerability to situational loneliness, whereas social support and sleep may buffer this effect. Loneliness, often conceptualized as a form of social pain, engages neural and physiological processes similar to those involved in physical pain and stress responses (Cacioppo et al., 2010; Eisenberger and Lieberman, 2004), contributing to heightened psychological and physiological vulnerability. Individuals experiencing loneliness tend to exhibit increased stress reactivity, including elevated anxiety, fear of negative evaluation, and depressive symptoms, while also showing reduced resilience factors such as optimism and self-esteem (Cacioppo et al., 2006; Hawkley and Cacioppo, 2010). In the context of social conflict, lonely individuals may perceive such situations as more threatening to their already limited social resources, thereby amplifying stress responses (Hawkley and Cacioppo, 2010). This dynamic reflects a self-reinforcing cycle, in which heightened stress further exacerbates feelings of social isolation. Consistent with this framework, longitudinal evidence indicates that increased loneliness is associated with a higher risk of chronic pain, greater pain-related interference, and a broader distribution of pain over time, whereas more diverse social engagement is linked to reduced loneliness and, indirectly, lower levels of chronic pain (Lee et al., 2023). Conversely, social support may buffer these effects by reducing perceived stress and promoting positive affect, thereby enhancing emotional wellbeing (Acoba, 2024). Taken together, these findings emphasize that social conflict exerts a profound impact on both emotional and physiological systems and underscore the importance of addressing loneliness and strengthening social support as key targets for mitigating stress in socially challenging contexts.

The attenuation of the initially significant sex difference in stress from baseline to the conflict condition appears to be driven by a critical interaction between sex and age, rather than the absence of a true sex effect. At baseline, men exhibited higher stress levels than women (Table 2); however, this difference became non-significant under the conflict condition, reflecting a shift in the underlying pattern of stress responses. Analyses revealed a significant sex × age interaction, indicating that stress changes differently with age for men and women (Figure 2). Specifically, stress tends to decrease with age in women, whereas it increases in men. This age-related decrease in stress observed in women is consistent with prior research demonstrating reduced psychophysiological stress reactivity in older adults compared to younger adults (Mikneviciute et al., 2023). In that study, older individuals exhibited lower subjective stress, attenuated cortisol responses, and reduced heart rate reactivity under acute stress conditions, suggesting an age-related dampening of stress responses. This opposing pattern suggests that the effect of sex on stress is not a simple main effect but depends on age, particularly under socially stressful conditions. As a result, variability within the same condition increased, with older women showing lower stress and older men showing higher stress. This led to greater overlap in stress distributions between men and women in the conflict condition, making the overall sex difference less clear and reducing its statistical significance. From a statistical perspective, this interaction indicates that the effect of sex on stress is conditional upon age, rendering the main effect less informative. These findings suggest that sex differences in stress are age-dependent rather than uniform across the lifespan, highlighting the importance of considering interaction effects rather than relying solely on main effects in psychophysiological research on social conflict.

The present study demonstrates that age-related emotional processes follow distinct, sex-specific trajectories, reflecting a form of functional specialization rather than a uniform pattern of aging. Specifically, aging appears to serve a stress-buffering role in women, whereas in men it is associated with enhanced engagement, reflected in concurrent increases in stress, happiness, and empathy under conflict. This high-arousal engagement pattern suggests that older men do not merely tolerate interpersonal challenges but actively transform them into opportunities for psychological reward and generativity. Such findings align with the concept of affect optimization, whereby individuals integrate positive and negative experiences to support adaptive functioning (Labouvie-Vief and Medler, 2002), and are further supported by recent evidence demonstrating age- and sex-specific differences in how emotion regulation modulates the impact of chronic stress, suggesting that men may exhibit greater variability in stress-related outcomes in later life depending on their regulatory capacities (Novotný et al., 2024). In line with this interpretation, regression analyses indicated that happiness during the conflict condition was negatively predicted by pre-experimental depression (assessed using PHQ-9) and positively predicted by emotional bank account and number of family members (*R*^2^ = 0.416, adjusted *R*^2^ = 0.388, *F* = 14.501, *p* < 0.001). Specifically, the positive upward trajectory of empathy in older men (*B* = 0.547) supports recent meta-analytic evidence that emotional empathy tends to increase across adulthood (Jarvis et al., 2024). For older men, heightened emotional resonance may serve as a tool to derive competence and meaning from social challenges, reflecting a sophisticated mode of regulation that extends beyond the simple avoidance of negative affect.

In contrast, women exhibited a “downward stabilization” pattern, characterized by a significant decline in stress during conflict (*B* = –0.888, *p* = 0.036). Consistent with Socioemotional Selectivity Theory (SST), older women appear to prioritize internal emotional harmony and minimize the impact of high-arousal negative stimuli (Carstensen and Mikels, 2005), consistent with recent findings indicating sex-specific patterns in emotion regulation and stress responses across aging, with women showing relatively stable responses under stress (Novotný et al., 2024). While women maintained higher overall empathy than men, age was negatively associated with empathy during conflict (*B* = –0.320), suggesting that the resources required to maintain internal stability may be prioritized over outward empathic engagement. In this context, emotional regulation in women may involve selectively attenuating empathic engagement as a strategy for self-preservation, which may help buffer against the physiological and psychological costs of conflict. Taken together, these findings underscore the necessity of a sex-specific perspective on successful aging. While aging is generally associated with enhanced emotional capacity, particularly empathy (Jarvis et al., 2024), the manifestation of these capacities depends on the interaction between biological sex, contextual demands, and available emotional resources. In particular, happiness under conflict appears to be shaped not only by reduced depressive burden but also by social (family structure) and affective (emotional bank account) resources. Consequently, interventions may benefit from being tailored to these distinct trajectories—specifically, the downward stabilization pattern observed in women and the high-arousal engagement pattern observed in men. For women, this may involve fostering emotional vitality and psychophysiological alignment, whereas for men, this may involve promoting meaningful, challenge-based engagement that may facilitate emotional investment and integrated social functioning across the lifespan.

The present findings indicate that exposure to social conflict is characterized by autonomic imbalance, reflected in reduced parasympathetic activity and a relative shift toward sympathetic predominance arising from parasympathetic withdrawal rather than an absolute increase in sympathetic activity. Specifically, lnHF was lower in the conflict condition, and RMSSD—a well-established marker of parasympathetic activity strongly correlated with HF-HRV (Kleiger et al., 2005)—negatively predicted stress, supporting evidence that greater parasympathetic activity is associated with improved emotional regulation and attenuated stress responses (Porges, 2001; Porges, 2007; Thayer and Lane, 2000; Thayer and Lane, 2009). Recent evidence further suggests that parasympathetic dominance is linked to adaptive affective functioning, including enhanced emotional attunement and reduced sympathetic reactivity, particularly in women, whereas sympathetic activation is more strongly associated with both positive and negative affect in men, supporting a two-dimensional autonomic space model in which sympathetic and parasympathetic systems may operate independently rather than as a simple reciprocal balance (Menashri Sinai et al., 2024). These findings suggest that individuals with greater parasympathetic regulation are better equipped to manage stress in socially challenging contexts, highlighting the role of parasympathetic functioning in recovery and resilience, as well as the potential benefits of interventions such as mindfulness-based stress reduction (Kleiger et al., 2005). Recent evidence further indicates that non-invasive vagal neuromodulation approaches, including HRV biofeedback and auditory-based interventions such as the Safe and Sound Protocol, can enhance vagal tone, improve autonomic balance, and facilitate emotional regulation and resilience through mechanisms such as baroreflex strengthening and increased parasympathetic activation (Gitler et al., 2025).

In contrast, lnLF did not differ between conditions, suggesting lower sensitivity to acute social conflict at the mean level. However, regression analyses revealed that higher LF-HRV was positively associated with stress, whereas RMSSD was negatively associated, indicating that autonomic imbalance characterized by reduced parasympathetic regulation and a relative shift toward sympathetic predominance may exacerbate stress responses, whereas greater parasympathetic activity may buffer stress responses. This interpretation is consistent with prior literature reporting mixed physiological contributions of LF-HRV (Quigley et al., 2024) and supports the view that the LF band should not be interpreted as a pure index of sympathetic activity. Rather, under psychological stress, alterations in LF-related indices may reflect complex interactions between sympathetic and parasympathetic influences and, in the present study, are most parsimoniously interpreted as a consequence of parasympathetic withdrawal rather than an absolute increase in sympathetic activation. Recent evidence further indicates that multiple HRV indices—including RMSSD, LF/HF ratio, and HF power—are significantly associated with psychological stress across both experimental and real-life contexts, thereby reinforcing the validity of HRV as a marker of stress-related autonomic imbalance (Immanuel et al., 2023). Taken together, these findings indicate that stress responses to social conflict arise from the combined effects of reduced parasympathetic regulation, a relative shift toward sympathetic predominance secondary to parasympathetic withdrawal, and psychological vulnerability, underscoring the importance of integrative approaches targeting both autonomic and psychological mechanisms to enhance emotional resilience and overall wellbeing.

A central finding of the present study is the dissociation between absolute and normalized indices of HRV, highlighting distinct influences of age and biological sex on autonomic regulation. While sex did not significantly influence absolute parasympathetic activity (lnHF) after controlling for age, it emerged as a significant predictor of normalized indices (HF norm, LF norm) and the LF/HF ratio, particularly under the conflict condition. This pattern suggests that age and sex exert differential effects depending on how HRV is operationalized. Specifically, lnHF reflects the overall magnitude of parasympathetic activity, which declines with advancing age, as evidenced by the significant negative association between age and lnHF at baseline (*B* = -0.033, *p* = 0.019), consistent with prior findings on age-related reductions in vagal tone (Choi et al., 2020; Olivieri et al., 2024). When age is included as a covariate, it accounts for a substantial proportion of variance in absolute HRV, thereby obscuring potential sex differences.

In contrast, normalized indices revealed robust sex differences independent of age, with women exhibiting higher HF norm (greater parasympathetic dominance) and men showing higher LF norm and LF/HF ratios, indicating a relative shift toward sympathetic predominance secondary to reduced parasympathetic activity rather than an absolute increase in sympathetic activity, across both baseline and conflict conditions. Importantly, these differences were not moderated by age, suggesting that they reflect intrinsic sex-related patterns of autonomic regulation rather than demographic variation. This divergence arises from the mathematical properties of normalization, which remove global age-related reductions in signal amplitude and instead capture the relative balance of autonomic activity. Collectively, these findings indicate that absolute HRV indices primarily reflect age-related physiological capacity, whereas normalized indices provide insight into sex-specific patterns of relative autonomic allocation and regulatory balance, particularly under stress.

Accordingly, normalized measures revealed a reciprocal pattern of autonomic organization, with women showing greater parasympathetic dominance (higher HF norm, lower LF/HF ratio) and men showing a relative shift toward sympathetic predominance secondary to reduced parasympathetic regulation (higher LF norm). Notably, these sex differences were most pronounced under the conflict condition. While baseline differences in LF/HF ratio were marginal, they became statistically significant during conflict (*F* = 5.712, *p* = 0.020), with men exhibiting higher LF/HF ratios than women, indicating a relative shift in autonomic balance toward sympathetic predominance rather than an absolute increase in sympathetic activity under stress. In contrast, women maintained higher relative parasympathetic engagement, consistent with prior evidence suggesting greater autonomic flexibility in women (Koenig and Thayer, 2016) and greater sympathetic reactivity in men under psychosocial stress (Kajantie and Phillips, 2006). This pattern is further supported by recent neurophysiological evidence demonstrating that women exhibit higher resting HRV—indicative of parasympathetic dominance—and may be more effective in modulating positive emotional valence (Min et al., 2023).

Importantly, age did not significantly influence LF/HF ratio or moderate its relationship with sex, further supporting the interpretation that these effects are sex-dependent rather than age-driven. Taken together, these findings demonstrate that absolute and normalized HRV indices capture distinct dimensions of autonomic function: lnHF reflects age-dependent parasympathetic capacity, whereas normalized indices reflect sex-dependent patterns of relative autonomic allocation and regulatory balance that become particularly pronounced under emotional stress. This distinction underscores the importance of integrating both absolute and normalized HRV measures to achieve a comprehensive understanding of autonomic regulation, as reliance on a single metric may obscure critical dimensions of physiological function.

The present findings provide strong evidence for a cognitive–physiological dissociation in women’s stress responding, that is, a mismatch between what individuals report feeling and how their bodies respond physiologically, as reflected in autonomic responses (e.g., greater relative parasympathetic predominance indexed by higher HF norm) and somatic responses (e.g., increased pain sensitivity). This pattern indicates a divergence between subjective experience and physiological outcomes. Age-adjusted analyses showed that men reported higher stress than women at baseline (*p* = 0.037), although this difference was attenuated during the conflict condition (*p* = 0.087). Critically, significant sex × age interactions were observed at both baseline (*p* = 0.018) and during the conflict condition (*p* = 0.033). Follow-up regression analyses revealed divergent trajectories, such that stress decreased with age in women (*B* = -0.888, *p* = 0.036) but increased with age in men. Despite this reduction in subjective stress among women, female sex emerged as a strong predictor of pain, and women exhibited higher post-conflict pain, highlighting a clear dissociation between perceived stress and physiological burden. Moreover, women’s autonomic responses (higher HF norm, indicating greater relative parasympathetic predominance) were aligned with their lower reported stress, whereas their somatic responses (increased pain sensitivity) were not, suggesting that sex differences in stress responding operate across multiple levels—subjective, autonomic, and somatic—rather than reflecting a unified process.

This dissociation suggests that emotional regulation may be achieved through partially independent systems. In women, reduced subjective stress with age likely reflects enhanced cognitive regulation, whereas higher HF norm indicates greater relative parasympathetic predominance during conflict rather than an absolute increase in parasympathetic activity. Rather than simply indicating that the body has returned to a relaxed state, this pattern suggests that the body is actively working to regulate emotions and maintain stability (Watanabe et al., 2026). Accordingly, these findings suggest that effective emotional regulation at the subjective level may entail hidden physiological costs. In particular, the coexistence of lower perceived stress and higher pain responses indicates that maintaining emotional stability may require sustained autonomic engagement, potentially increasing both interoceptive sensitivity and somatic reactivity. In this context, higher HF norm may reflect ongoing relative parasympathetic predominance and regulatory modulation rather than recovery *per se* (Appelhans and Luecken, 2006; Laborde et al., 2017; Porges, 2007; Thayer and Lane, 2009; Watanabe et al., 2026). This interpretation is further supported by the Vagal Tank Theory, which conceptualizes cardiac vagal control as a dynamic self-regulatory resource operating across three functional stages—resting, reactivity, and recovery—suggesting that elevated vagal indices during stress may reflect the active mobilization and utilization of regulatory capacity rather than a return to baseline recovery (Laborde et al., 2018; Prell et al., 2025). Thus, emotional calmness should not be interpreted as the absence of physiological stress, but rather as a state that may coexist with continued physiological processing demands. Taken together, these findings underscore the importance of a multi-level framework in stress research, as reliance on self-reported measures alone may underestimate physiological strain, particularly in women.

The design comparing baseline and conflict conditions in this study represents a methodologically sound approach. Even seemingly neutral social scenes (e.g., everyday situations) may evoke emotional responses due to their association with individuals’ personal memories or social experiences, making it difficult to ensure true emotional neutrality when such stimuli are used as controls. In contrast, employing a fixation cross as the baseline provides a clear reference point for assessing physiological and subjective changes under conditions of minimal emotional engagement. Moreover, given that not all participants may uniformly perceive the images as “conflictual,” the inclusion of the phrase “social conflict” during stimulus presentation serves as a useful strategy to clarify the intended meaning of the stimuli and enhance interpretive consistency across participants. Importantly, rather than examining psychological or physiological responses separately, the present study analyzed both types of responses together within the conflict condition, allowing for a more complete understanding of how individuals react to social conflict. Using regression analyses, key predictors of stress and pain—such as autonomic activity, loneliness, and psychological vulnerability—were identified. This multi-level analytical approach, which integrated subjective measures with both absolute and normalized HRV indices, provides a more comprehensive understanding of responses to social conflict by capturing how individuals think, feel, and physiologically respond in an integrated manner. Furthermore, the findings underscore the methodological importance of interpreting relative autonomic patterns in conjunction with absolute autonomic activity, as reliance on either metric alone may obscure critical dimensions of stress-related physiological regulation. This approach also offers meaningful implications for the development of interventions aimed at mitigating stress and pain in socially challenging contexts.

**FIGURE 4:**
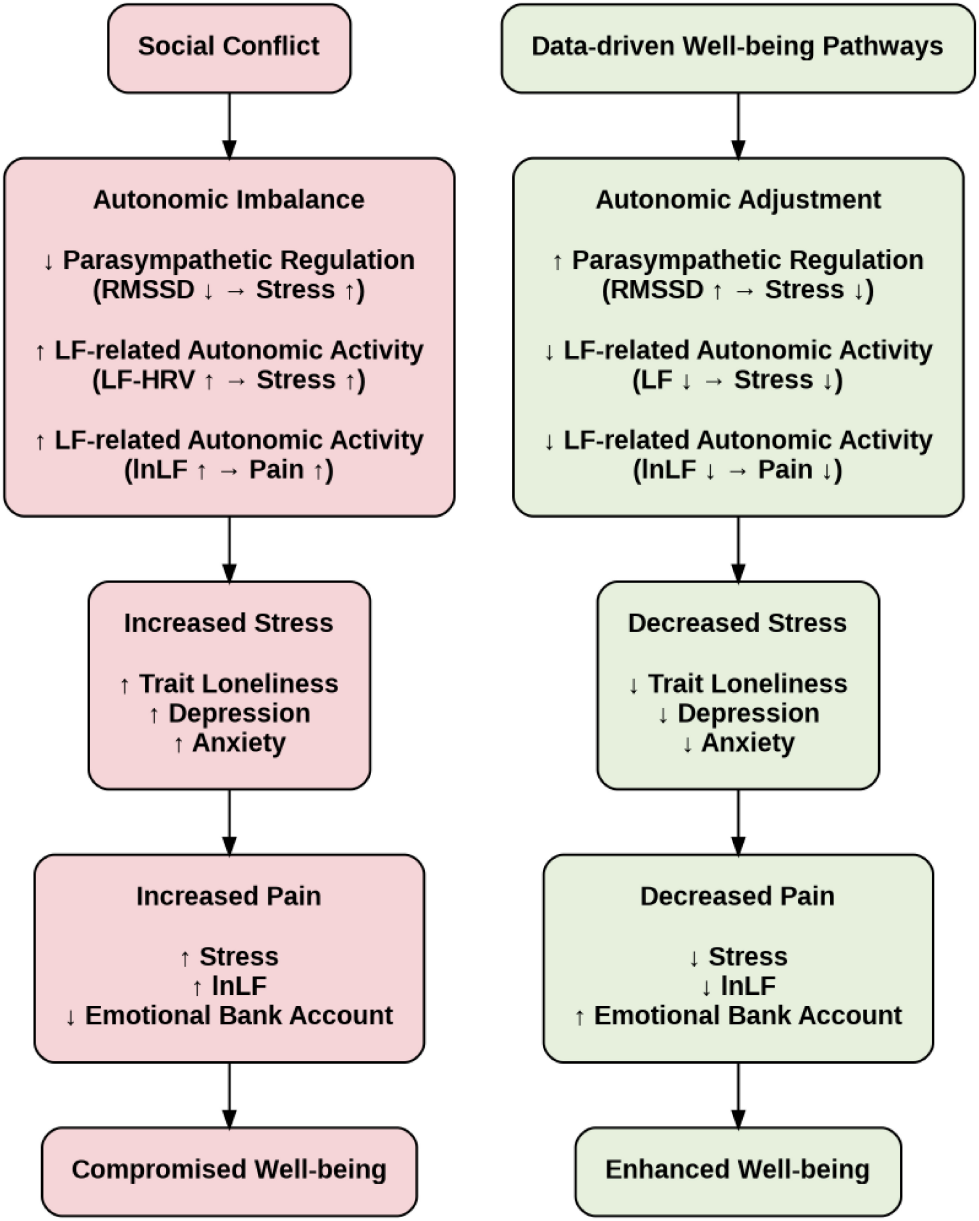
Risk and resilience pathways of wellbeing. Left panel (risk pathway: social conflict → compromised wellbeing): Exposure to social conflict images triggered a cascade of psychological distress accompanied by autonomic dysregulation. The principal physiological alteration was a reduction in parasympathetic regulation, reflected by lower RMSSD and HF power, indicating selective parasympathetic withdrawal during conflict exposure. In contrast, absolute LF power did not significantly change. Therefore, elevations in LF-related indices should not be interpreted as evidence of pure sympathetic activation. Rather, under conditions in which overall autonomic capacity remained within a near-normal range, selective parasympathetic withdrawal altered the relative spectral distribution, resulting in greater relative LF predominance. This physiological dysregulation was further amplified by pre-existing psychological vulnerabilities, including trait loneliness, depression, and anxiety. Under these conditions, increased stress, together with elevations in LF-related indices and depletion of emotional resources (emotional bank account), was associated with amplified pain perception. Sex and age further modulated this cascade, contributing to individual differences in vulnerability and adaptation. Collectively, this sequence—from selective parasympathetic withdrawal and relative spectral redistribution to stress and pain—represents a risk pathway leading to compromised wellbeing. Right panel (resilience pathway: autonomic adjustment → enhanced wellbeing): The right panel depicts a data-driven resilience pathway in which improved autonomic regulation buffers individuals against conflict-induced distress. This adaptive pathway is characterized by improved parasympathetic regulation, reflected by higher RMSSD, which limits the escalation of stress. Under conditions of stabilized absolute autonomic capacity, reductions in LF-related indices should likewise not be interpreted as simple sympathetic withdrawal. Instead, they represent a healthier redistribution of autonomic resources and restoration of autonomic balance. By maintaining both the quantity of autonomic resources (absolute spectral power) and the quality of their allocation (relative spectral composition), the system attenuates the reciprocal amplification of stress and pain. These adaptive physiological processes are associated with lower pain and greater emotional resources, reflecting emotional replenishment and resilience. Furthermore, happiness during conflict was associated not only with lower depressive burden but also with affective and social resources, including emotional investment and family-based support. Thus, wellbeing under social conflict appears to depend not merely on reducing vulnerability but also on preserving autonomic capacity and actively maintaining psychosocial resources. Overall, this pathway represents a protective mechanism in which autonomic adjustment, together with emotional and social resources, promotes resilience and enhanced wellbeing.

Unlike previous studies that focused primarily on autonomic responses to either social pain or physical pain (Jang et al., 2024), the present study demonstrates that social conflict imagery concurrently evokes social pain (loneliness), emotional distress, and physical pain within a single experimental framework. Moreover, the inclusion of HRV-derived indices, together with analyses of sex and age effects, provides novel insights into individual variability in autonomic regulation, with women exhibiting greater parasympathetic predominance and men showing a relative shift toward sympathetic predominance secondary to parasympathetic withdrawal rather than an absolute increase in sympathetic activity. Importantly, our conceptual framework (Figure 4) suggests that social conflict induces autonomic imbalance, reflected by reduced parasympathetic regulation (RMSSD ↓ and lnHF ↓) and a consequent relative shift toward sympathetic predominance, which contributes to heightened stress and pain, particularly among psychologically vulnerable individuals. In contrast, greater parasympathetic regulation and preservation of autonomic balance were associated with lower stress, reduced pain, and greater emotional resources. Taken together, these findings suggest that autonomic dynamics serve as key mechanisms linking physiological regulation, psychological vulnerability, and resilience, providing a unified psychophysiological framework for understanding responses to social conflict.

Several limitations of the present study should be acknowledged. First, the acute experimental design precludes conclusions regarding the cumulative effects of chronic exposure to social conflict stimuli and the long-term consequences of repeated stress responses (McEwen, 1998). Second, the sample was drawn primarily from South Korea and concentrated in the 40–60 age range, limiting generalizability to other cultures and age groups. Third, pain assessment relied on a single mechanical stimulus, although pain is inherently multidimensional and may differ across sensory modalities (Hastie et al., 2005; Weaver et al., 2021). Finally, hormonal factors, such as menstrual cycle phase and hormone levels, were not assessed despite their potential influence on HRV and pain responses (de Jager et al., 2026; Schmalenberger et al., 2020). Taken together, these limitations highlight the need for future research employing longitudinal designs, more diverse and lifespan-inclusive samples, multimodal pain assessments, and hormonal measurements to enhance our understanding of the psychophysiological processes underlying social conflict and to improve the robustness and generalizability of the present findings.

## 5 Conclusion

In conclusion, the present study demonstrates that exposure to social conflict induces a coordinated pattern of psychological distress and autonomic dysregulation, characterized by increased negative affect and pain alongside reduced parasympathetic activity. Importantly, these responses were not uniform but varied according to sex and age, with women showing greater parasympathetic dominance yet higher pain sensitivity, and divergent age-related trajectories observed between men and women. Beyond identifying a vulnerability pathway, the findings provide compelling evidence that the same physiological and psychological systems underlying stress and pain can be redirected toward resilience and wellbeing (Figure 4). Specifically, autonomic flexibility emerged as a central mechanism, whereby greater parasympathetic regulation and preservation of autonomic balance, rather than absolute reductions in sympathetic activity, were associated with improvements in stress, affect, and pain. Moreover, the observed increases in normalized LF-HRV in men are most appropriately interpreted as reflecting a relative shift toward sympathetic predominance secondary to parasympathetic withdrawal rather than an absolute increase in sympathetic activation. In parallel, psychological factors such as emotional resources and social cognition further shaped these responses, highlighting the interplay between physiological regulation and psychological resilience. These results suggest that stress responses are not fixed but modifiable and context-dependent, reflecting dynamic regulatory processes rather than static dysfunction. From a translational perspective, interventions that enhance parasympathetic regulation, strengthen emotional resources, and address psychological vulnerability may facilitate a shift from maladaptive stress amplification toward adaptive recovery and resilience. Ultimately, this bidirectional regulatory framework reframes stress-related responses as flexible systems that, when properly regulated, can promote adaptation, recovery, and sustained psychological wellbeing.

## Data Availability

The data that support the findings of this study are available from the corresponding authors upon reasonable request.
